# Gut microbiome modulates tacrolimus pharmacokinetics through the transcriptional regulation of ABCB1

**DOI:** 10.1186/s40168-023-01578-y

**Published:** 2023-07-06

**Authors:** Alexandra L. Degraeve, Vincent Haufroid, Axelle Loriot, Laurent Gatto, Vanessa Andries, Lars Vereecke, Laure Elens, Laure B. Bindels

**Affiliations:** 1 Department of Integrated PharmacoMetrics, PharmacoGenomics and PharmacoKinetics, Louvain Drug Research Institute, Université catholique de Louvain, Brussels, Belgium; 2 Metabolism and Nutrition Research Group, Louvain Drug Research Institute, Université catholique de Louvain, Brussels, Belgium; 3 Louvain centre for Toxicology and Applied Pharmacology, Institut de Recherche Expérimentale et Clinique, Université catholique de Louvain, Brussels, Belgium; 4 Department of Clinical Chemistry, Cliniques Universitaires Saint-Luc, Brussels, Belgium; 5 Computational Biology and Bioinformatics Unit (CBIO), de Duve Institute, Université catholique de Louvain, Brussels, Belgium; 6 Department of Internal Medicine and Pediatrics, Ghent University, Ghent, Belgium; 7 VIB-UGent Center for Inflammation Research, Ghent, Belgium; 8 Ghent Gut Inflammation Group (GGIG), Ghent, Belgium; 9 WELBIO department, WEL Research Institute, Wavre, Belgium

**Keywords:** Inter-individual pharmacokinetic variability, Intra-individual pharmacokinetic variability, Cytochrome P-450 CYP3A, Narrow therapeutic index, Therapeutic drug monitoring, Immunosuppressive therapy

## Abstract

**Background:**

Following solid organ transplantation, tacrolimus (TAC) is an essential drug in the immunosuppressive strategy. Its use constitutes a challenge due to its narrow therapeutic index and its high inter- and intra-pharmacokinetic (PK) variability. As the contribution of the gut microbiota to drug metabolism is now emerging, it might be explored as one of the factors explaining TAC PK variability. Herein, we explored the consequences of TAC administration on the gut microbiota composition. Reciprocally, we studied the contribution of the gut microbiota to TAC PK, using a combination of in vivo and in vitro models.

**Results:**

TAC oral administration in mice resulted in compositional alterations of the gut microbiota, namely lower evenness and disturbance in the relative abundance of specific bacterial taxa. Compared to controls, mice with a lower intestinal microbial load due to antibiotics administration exhibit a 33% reduction in TAC whole blood exposure and a lower inter-individual variability. This reduction in TAC levels was strongly correlated with higher expression of the efflux transporter *ABCB1* (also known as the p-glycoprotein (P-gp) or the multidrug resistance protein 1 (MDR1)) in the small intestine. Conventionalization of germ-free mice confirmed the ability of the gut microbiota to downregulate *ABCB1* expression in a site-specific fashion. The functional inhibition of ABCB1 in vivo by zosuquidar formally established the implication of this efflux transporter in the modulation of TAC PK by the gut microbiota. Furthermore, we showed that polar bacterial metabolites could recapitulate the transcriptional regulation of *ABCB1* by the gut microbiota, without affecting its functionality. Finally, whole transcriptome analyses pinpointed, among others, the Constitutive Androstane Receptor (CAR) as a transcription factor likely to mediate the impact of the gut microbiota on *ABCB1* transcriptional regulation.

**Conclusions:**

We highlight for the first time how the modulation of *ABCB1* expression by bacterial metabolites results in changes in TAC PK, affecting not only blood levels but also the inter-individual variability. More broadly, considering the high number of drugs with unexplained PK variability transported by ABCB1, our work is of clinical importance and paves the way for incorporating the gut microbiota in prediction algorithms for dosage of such drugs.

Video Abstract

**Supplementary Information:**

The online version contains supplementary material available at 10.1186/s40168-023-01578-y.

## Background

In kidney transplantation, lifelong immunosuppressive therapy is required to prevent organ rejection [1]. Maintenance therapy typically consists of a triple-drug regimen including corticosteroids, a calcineurin inhibitor and an anti-metabolite [1] with the calcineurin inhibitor tacrolimus (TAC) considered a pillar of the current immunosuppressive strategy [2]. Despite its effectiveness, maintaining optimal levels of TAC is arduous because of its low therapeutic index. Additionally, the difficulty resides in predicting the ideal drug dosage to reach the desired therapeutic levels owing to huge inter- and intra-individual pharmacokinetic (PK) variability [3, 4]. Avoiding over- and under-dosage resulting in increased toxicity on the one hand and graft rejection on the other hand is essential to preserve a viable allograft and to avoid side effects [1].

After oral administration, TAC reaches the small intestine where the efflux transporter ABCB1 (ATP Binding Cassette Subfamily B Member 1, formerly known as P-gp, MDR1) limits its intestinal absorption [5]. In enterocytes and hepatocytes, cytochrome P450 (CYP) 3A isoenzymes are responsible for TAC extensive and highly variable pre-systemic metabolism [6], giving rise to at least 15 metabolites [7]. Eventually, those metabolites are excreted through the biliary route, and less than 0.5% of the parent drug is recovered unchanged in the faeces or urine [8].

Efforts in the field of pharmacogenetics highlighted the consequence of single nucleotide polymorphisms in TAC-processing transporter and enzyme genes on TAC PK inter-individual variability, leading to new genotype-based dosage recommendations (reviewed in Degraeve et al.) [9]. Independently from host genetics, TAC PK is also characterized by a significant intra-patient variability associated to drug levels escaping the therapeutic range [10] and identified as an important risk factor for graft rejection [4, 11]. These elements explain why TAC management in clinical practice is still challenging, highlighting the importance of identifying other sources of PK variability.

The importance of the gut microbiota for explaining the fate of immunosuppressive drugs in the organism is still largely understudied, although it is now widely accepted that the complex ecosystem of intestinal microbes dynamically takes part in drug metabolism [12]. Not only gut microorganisms express numerous enzymes able to directly metabolize xenobiotics [13, 14], but also they are able to influence the host PK phenotype through different indirect processes [15, 16]. Inter-individual differences in PK profiles might thus be explained by between-subject differential microbiota composition or function. On the other hand, contrary to the host genetic makeup, the microbiota is not inflexible and might thus also be a source of intra-individual variability.

Studies have highlighted differences in the gut microbiota composition of renal transplanted patients compared to healthy individuals [17, 18], with significant changes also observed in the microbiome of patients’ pre- compared to post-transplantation samples [19], indicating a potential interplay between microbiota, transplant status and immunosuppressive pharmacotherapy. Additional lines of evidence support our hypothesis of the existence and the likely importance of an interaction between immunosuppressive therapy and the gut microbiota. Indeed, case reports described alteration in TAC blood concentrations after diarrhoea episodes [20, 21] or even antibiotherapy [22, 23]. Moreover, a clinical pilot study showed that TAC dose escalation during the first month after transplantation was correlated with the initial abundance of the bacterium *Faecalibacterium prausnitzii* [24]. Subsequently, in vitro experiments highlighted that many bacteria from the *Clostridiales* order are able to metabolize TAC into an inactive metabolite through a C-9 keto-reduction of TAC [25].

In the current study, we aim at exploring (i) the consequence of TAC administration on the gut microbiota composition, and reciprocally (ii) the contribution of the gut microbiota to TAC PK, using a combination of in vitro and in vivo models.

## Methods

### In vivo studies

#### Animal strains and care

In all experiments, male C57Bl6 mice (7 weeks old, obtained from Janvier Laboratories) were used, except in the alternative antibiotic cocktail (ATB2) protocol where female BALB/c mice (5 weeks old, obtained from Charles River Laboratories) were used. Mice were kept in specific pathogen-free (SPF) conditions. Mice were co-housed (2–3 mice/cage) and maintained on a 12-h light–dark cycle with ad libitum access to water and standard chow diet (D12450Ji, Research Diets, USA). Body weight and food intake were monitored every 2 days. Several independent experiments were carried out and, for each experiment, mice were allocated into the different treatment groups based on their body weight.

For the conventionalization protocol, male C57Bl6 germ-free (GF) mice were born and raised at the Ghent Germ-free and Gnotobiotic mouse facility (Ghent University, *LA2400451*). They were maintained in a sterile environment under controlled conditions (10-h light–dark cycle) with ad libitum access to water and diet (2018S, Envigo, USA). GF mice were housed and bred in ‘open’ cages in positive-pressure GF isolators. Before colonization, GF mice were exported from isolators to positive-pressure isocages and left to acclimatize for several days. The conventionalisation protocol is described in the Supporting Information.

#### Tacrolimus administration

TAC was suspended in a mix of water and propylene glycol (90:10), 3 to 5 days before the first administration and kept at 4 °C. The stability of the solution was validated for 15 days. TAC was first dissolved in propylene glycol, then the water was added. The suspension was homogenized using an ultrasonic homogenizer 3 times for 15 s (QSonica Q700 sonicator, QSonica LLC., USA). TAC concentration was always checked before the first and after the last gavage to control for the dose. TAC or the vehicle was administered once a day (always at the same period) by oral gavage. A dose of 3 mg of TAC/kg of body weight was administered for 5 days, except in the dose determination study where different escalating doses (from 0.1 to 10 mg/kg of body weight) were tested (4 days of treatment). Mice were fasted for minimum 4 h before the last gavage because preliminary experiments demonstrated a reduced variability in TAC PK in fasted state (data not shown).

#### Antibiotic supplementation

The antibiotic (ATB) cocktail (adapted from Iida et al*.* [26]) was composed of neomycin (1 g/L), vancomycin (0.5 g/L) and meropenem (0.25 g/L). It was administered via drinking water and renewed every other day. In the adequate treatment groups, ATB administration started 4 days before the first gavage (TAC or vehicle) to ensure adequate microbial depletion and pursued during the treatment period. This ATB cocktail is the one used throughout the study, unless indicated otherwise.

The alternative antibiotic cocktail (ATB2) was composed of neomycin (0.5 g/L) and ampicillin (1 g/L). It was administered for 13 days via drinking water and renewed every other day.

#### Zosuquidar supplementation

Zosuquidar (ZSQ) was suspended in aqueous 0.5% methylcellulose (based on Matsuda et al. [27]). A single dose of 30 mg/kg of body weight was administered 15 min before last TAC administration by oral gavage (based on Kono et al. [28]).

#### Sample collection

Fresh faeces were collected and quickly frozen on dry ice before storage at − 80 °C until further analysis or use. Whole blood samples were collected from the tail of awake freely moving mice, at various time points (ranging from 1 to 24 h = T0, after last gavage). The blood sampling strategy used to generate the concentration–time curve of TAC blood levels in mice is detailed in the Supporting information. Before necropsy, mice were fasted for minimum 4 h and anesthetized with ketamine-xylazine or isoflurane. Hepatic and intestinal tissues were quickly collected and frozen in liquid nitrogen before storage at − 80 °C until further analysis.

### In vitro studies

#### Cell culture

Human embryonic kidney cells (HEK293 cells) and human epithelial colorectal adenocarcinoma cells (Caco-2 cells) were cultured in Dulbecco’s modified Eagle medium with high glucose and glutamine (Invitrogen, UK) supplemented with 10% (v/v) of foetal bovine serum and 1% (v/v) of antibiotic–antimycotic at 37 °C in the presence of 5% of CO_2_. Caco-2 cell culture medium was also supplemented with 1% non-essential amino acids. Additional experiments on human colon carcinoma (LS174T) cells, characterization of HEK293 transfected cell lines and ABCB1 functionality assay analyses are described in the Supporting information.

#### Faecal water preparation

Faecal water (FW) was freshly prepared from frozen faecal samples (protocol adapted from Pötgens et al. [29]). Briefly, faeces were diluted into phosphate-buffered saline (PBS) (ratio 1 mg:5μL) and homogenized 4 min at 30 Hz using a Tissue Lyser II (Qiagen, Germany). The homogenate was centrifuged (10 min, 10,000 g, 4 °C). The supernatant was centrifuged again (3 min, 2000 g, 4 °C) and the subsequent supernatant was used as FW.

#### Caco-2 cell treatment

Caco-2 cells were seeded in 6-well plates at a density of 1 × 10^6^ cells/well. After 48 h of growth, the medium was renewed and 10% of medium was replaced by either the FW from control mice (FWctl) or the FW from the same mice after 7 to 10 days of ATB-mediated microbial depletion (FWatb), or the ATB cocktail itself (ATB), or the vehicle (PBS) as control. After 48 h of exposure, cells were washed and collected for RNA extraction and subsequent gene expression analysis. Absence of cytotoxicity of the FW was confirmed using a mitochondrial activity assay (Cell Proliferation Reagent WST-1, Roche, Switzerland) following the manufacturer’s instructions.

### Tacrolimus assay

Blood quantification of TAC was performed on a high-performance liquid chromatography-tandem mass spectrometry (HPLC–MS/MS) method, as already described elsewhere [30] and further optimized for the small amounts of blood available in mouse PK studies. Briefly, the Agilent HPLC 1290 Infinity system is coupled to the 6460 Triple Quadrupole instrument. Fifty microlitres of whole blood was pre-treated and vortexed with 50 μl zinc sulfate 0.1 M, and 125 μl internal standard (ascomycin, 5 μg/L) in methanol. Samples were sonicated for 5 min, before 5 min of centrifugation at 13,500 rpm. Forty microlitres of the supernatant was injected into the system. The precolumn used was the 10 × 4 mm 3 μm Mercury Phenomenex, and the analytical column was the 4.6 × 50 mm 1.8 μm Agilent Zorbax Eclipse XDB C18, maintained at 60 °C. The mobile phase of pump 1 contained a mixture of H_2_O/methanol (40/60%), and pump 2 2 mM NH_4_^+^ acetate, and aqueous 0.5% formic acid/ methanol (5/95%). TAC retention time was 1.59 min. The method is fully validated in terms of analytical performance (limit of quantification: 0.5 ng/ml; imprecision < 5%) and ion suppression effects. The laboratory participates to the TAC International Proficiency Testing Scheme (ASI, London, UK).

### Pharmacokinetic analysis

All data generated in this project (*n* = 351 PK data) were used to develop a population PK model of TAC in mice at steady-state with an average number of observations per subject of 3.5. Population PK modeling of TAC blood concentrations was performed with MonolixSuite-2020R1 software (Lixoft, France) using the stochastic approximation expectation–maximization (SAEM) algorithm. Between-subject variability (eta) was described using an exponential model and covariance between random effects was investigated. Three error models (i.e. proportional, additive and combined) to describe the residual error were tested. The influence of mouse treatment on PK parameters was also investigated through its association with the eta of PK parameters. Additional information on the building of the population PK model (e.g. the precise description of number of measurements per time points) is provided in the Supporting Information.

A one-compartment model, first-order absorption and elimination with an additive error model best described the data. Covariance between random effects of all PK parameters was included and the group of treatment was identified as a significant covariate for the apparent clearance (CL/F) between-subject variability (*p* = 1.72 × 10^−6^). Conditional modes of individual population PK parameters (volume of distribution (V), absorption rate constant (Ka) and CL/F) were estimated using Markov chain Monte Carlo (MCMC) convergence assessment. From these primary PK parameters, individual values for the area under the curve (AUC_0-24 h_) and half-life (T½) were derived.

### Gut microbiota analysis

Genomic DNA was extracted from faeces using a QIAamp DNA Stool Mini Kit (Qiagen, Germany), including a bead-beating step. Absolute quantification of the total bacteria was performed by quantitative polymerase chain reaction (qPCR) using the primers Bacteria Universal P338f (ACTCCTACGGGAGGCAGCAG) and P518r (ATTACCGCGGCTGCTGG) [31]. 16S rRNA gene sequencing and ensuing bioinformatics and biostatistics analyses (including amplicon sequence variants (ASV) identification [32]) were performed as previously described [29]. Full details are provided in the Supporting Information.

### Gene expression analysis

#### qPCR analysis

Total RNA from mouse hepatic and intestinal tissues or from Caco-2 cells was isolated using the TriPure reagent (Roche, Switzerland). cDNA was obtained by reverse transcription of 1 μg of total RNA using the Goscript RT Mix OligoDT kit (Promega, the Netherlands). Real-time qPCR was performed with a CFX96 TouchTM instrument and software (Biorad, USA) using SYBR Green (Eurogentec, Belgium) for detection. All samples were run in duplicate in a single 96-well reaction plate, and data were analysed according to the 2^−ΔΔCT^ method. The purity of the amplified product was verified by analysing the melt curve performed at the end of amplification. The ribosomal protein L4 (*Rpl4*) was chosen as reference gene for all mouse tissues. The primer sequences for the targeted mouse genes are detailed in Table S1. The 18S ribosomal RNA (*18S rRNA*) was chosen as reference gene for human cell analyses. The primer sequences for the targeted human genes are detailed in Table S2.

#### Cellular whole transcriptome analysis

Caco-2 RNA samples were sequenced for a whole transcriptome analysis using a 2 × 150 paired-end configuration on a NovaSeq 6000 instrument (Macrogen, Netherlands). Raw sequence data generated from Illumina TruSeq Stranded library were processed using a standard RNAseq pipeline. The Supporting Information describes in full detail the sample preparation, sequencing method, bioanalysis workflow and biostatistics.

### Statistical analysis

Data are presented as mean ± SEM or as whiskers plots with minimal and maximal values. Data distributions were inspected for normality using the Shapiro–Wilk normality test while outliers were checked using the Grubbs test and removed (*α* = 0.05). For normally distributed data, Student’s *t*-test (two groups) or one-way ANOVA with Bonferroni post hoc tests (more than two groups) was applied to evaluate the statistical significance of differences between groups. For repeated measures, a repeated measures ANOVA with Bonferroni post hoc tests was used. Equality of variance was checked with either Fisher’s exact test (two groups) or Brown-Forsythe’s test (more than two groups). When variance equality was not verified, a Welch’s* t* test or a Welch’s ANOVA with Dunnett post hoc tests was applied (for two groups or more than two groups, respectively). For non-normally distributed data, a Mann–Whitney test (two groups) or a Kruskal–Wallis test with Dunn’s post hoc tests (more than two groups) was applied to evaluate the statistical significance of differences between groups. A two-way ANOVA followed by Bonferroni post hoc tests was used to assess the significance of the influence of two independent variables on one dependent variable.

To test the association between two continuous variables, a Pearson’s correlation test was used for normally distributed data. In other cases, Spearman’s correlation test was applied. Detailed statistical analyses of the microbiota and the whole transcriptome dataset are described in the Supporting Information. In any case, *p* < 0.05 was considered statistically significant. Statistical analyses were carried out using GraphPad Prism v9.1.2 for windows (GraphPad Software, USA) and R.

## Results

### Establishment of a PK model of oral TAC administration in mice

**Fig. 1:**
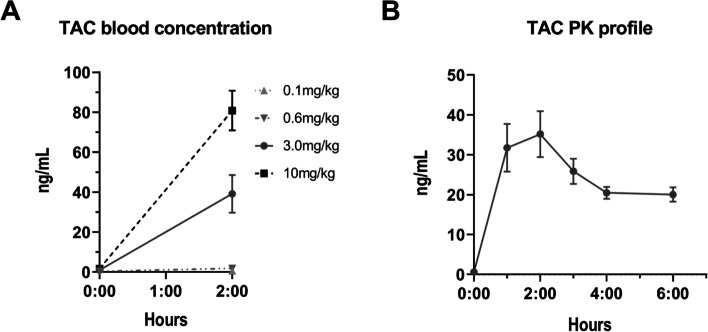
Determination of tacrolimus (TAC) pharmacokinetics (PK) profile in mice. **A** Blood concentration of TAC according to the dose (*n* = 10/group). **B** TAC blood concentration–time curve at the selected dose of 3 mg/kg (24 mice providing each 3 measurements, *n* = 12 measurements/time points covering 6-time points, as described in the Supplementary Methods)

One of the challenges associated with mouse models of TAC PK is their lack of reproducibility that may arise, among others, from the poor water solubility of TAC. Thus, we first implemented a robust, clinically relevant, mouse model of TAC PK. An optimized suspension for TAC administration in mice was obtained with a mix of water and propylene glycol (90:10). We administered TAC solution to mice by oral gavage at different concentrations (ranging from 0.1 to 10 mg/kg) to select an appropriate dosage. In human population, the absorption peak is reached rapidly, around 2 h after the drug intake, with blood concentrations ranging from 10 to 40 ng/mL [3, 33]. With a dose of 3 mg/kg, similar levels were obtained at 2 h after the last gavage with an average of 39.1 ng/mL (Fig. 1A). Moreover, the PK data were best described with a one-compartment model with first-order absorption and elimination which fits with most of human models [34, 35]. For all these reasons, a dose of 3 mg/kg of body weight was further selected for the rest of the experiments.

With this dose, we generated a full concentration–time curve of TAC blood levels at steady-state (Fig. 1B). The steady-state of a drug is achieved when the rate of input is equal to the rate of elimination, universally considered as equivalent to 5 times the half-life (corresponding for TAC to ~ 60 h in human [36]). Blood levels at T0h and T2h were reproducible with the previous experiment and the whole profile confirmed an absorption peak at 2 h after the last administration. A mono-compartmental population PK model of TAC blood concentrations in mice was developed by pooling all the available PK data in order to derive individual primary (CL/F, V and Ka) and secondary (AUC_0–24 h_ and T½) PK parameters (Table 1).

**Table 1:** Tacrolimus (TAC) population pharmacokinetics (PK) model

TAC PK model		Mean	IC 95%
**Primary PK parameters**	Cl/F	0.32 L/h	[0.29–0.35]
	V	1.88 L	[1.68–2.08]
	Ka	1.20 h^−1^	[1.08–1.32]
**Derived PK parameters**	AUC_0-24 h_	274.4 ng h/mL	[242.2–306.5]
	T½	4.08 h	[3.92–4.23]

TAC primary PK parameters (oral clearance (Cl/F), volume of distribution (V), absorption rate constant (Ka)) generated from a mono-compartmental model and derived PK parameters (area under the curve (AUC_0-24 h_) and half-life (T ½)) (*n* = 351 blood samples obtained in 99 mice, as described in the ‘Pharmacokinetic analysis’ subsection of the ‘Methods’ section)

Altogether, our results demonstrate that the established TAC PK model is robust, expandable to humans and, therefore, clinically relevant.

### Gut microbiota composition evolves upon TAC treatment

To evaluate the potential impact of TAC oral administration on the gut microbiota composition, we compared the faecal bacterial composition of mice treated with TAC (TAC) or with the vehicle (CTL). Faeces were collected at baseline (day 0) and after 2 and 5 days of treatment.

At the ASV level, we compared differences in the α-diversity induced by TAC treatment using different computed indexes. As observed in Figure S1, TAC treatment induced a significant reduction in the evenness of the gut microbiota as measured by Simpson’s and Heip’s evenness indexes after 5 days of TAC oral administration when compared to vehicle-treated mice. TAC-treated mice had also significantly reduced indexes combining richness and evenness (Shannon and Simpson indexes) whereas TAC administration did not affect the richness when considered solely (measured using Chao1 and Observed ASV indexes).

**Fig. 2:**
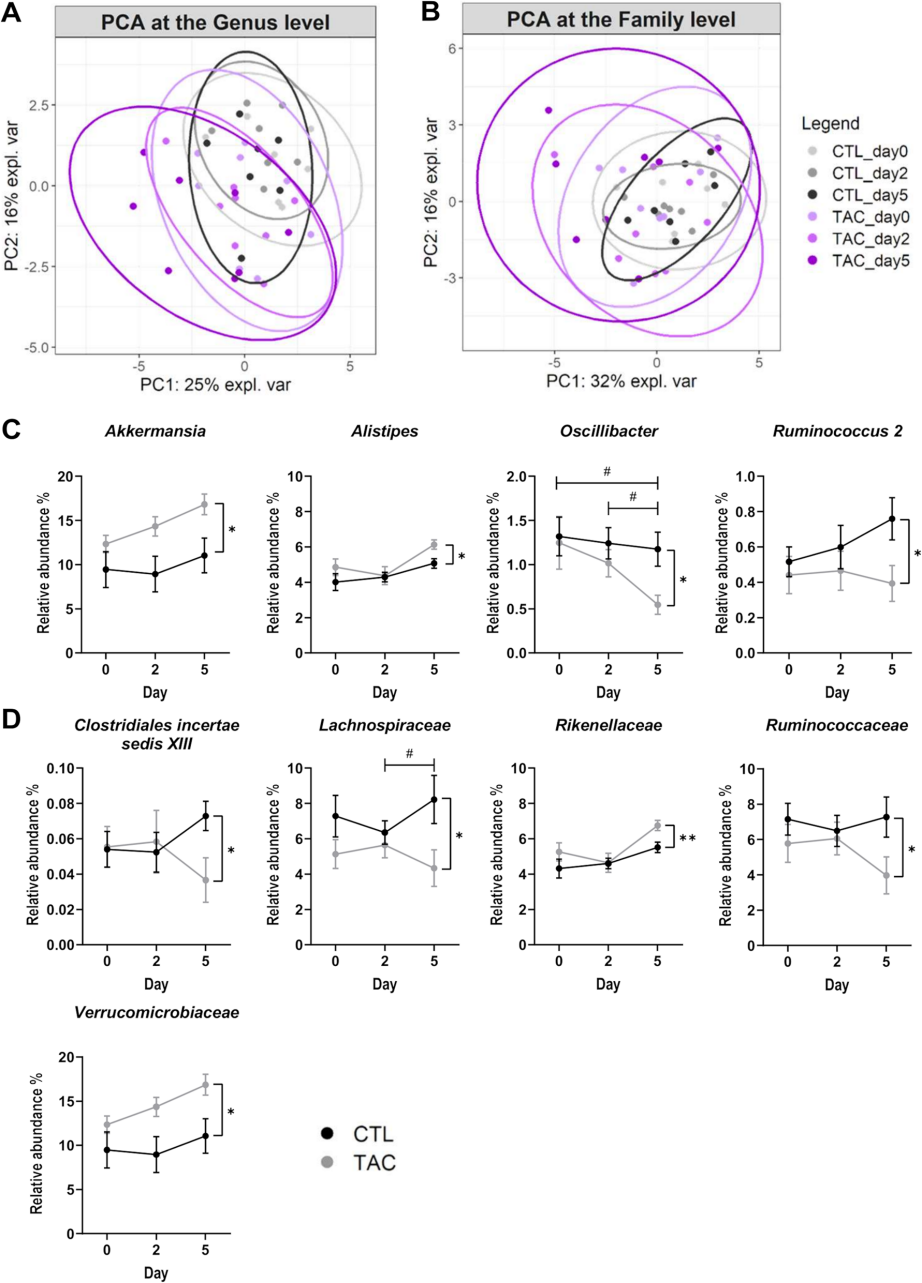
Faecal microbiota composition evolves upon tacrolimus (TAC) treatment. **A**, **B** Principal component analysis (PCA) of the relative abundances of taxa in control (CTL) and TAC-treated mice. Data are presented for the genus **A** and the family **B** taxonomic levels before and after 2 and 5 days of oral gavage. **C**, **D** Relative abundance of genera (**C**) and families (**D**) identified as significantly impacted by TAC treatment (*n* = 7–8/group). **p < 0.01, *p < 0.05 (Mann–Whitney, CTL vs TAC). ^#^*p* < 0.05 (Friedman test with Dunn post hoc test, TAC repeated measures)

As depicted on the principal component analysis (PCA) plots looking at the β-diversity, all mice had similar microbiota composition at baseline, both at the genus and family levels (Fig. 2A, B). After 5 days of TAC treatment, the gut microbiota was significantly affected at the genus level and results indicated that TAC administration accounted for 15% of the variability in the dataset (PERMANOVA, 1000 permutations, *p* < 0.05). We did not observe any significant change in the total bacterial load of both CTL and TAC mice over time (Figure S2).

Next, we identified the taxa for which the relative abundance was significantly impacted by 5 days of TAC administration (Fig. 2C, D, Figure S3). At the genus level, the relative abundance of *Akkermansia* and *Alistipes* were increased under TAC treatment, whereas the relative abundance of *Oscillibacter* and *Ruminococcus 2* were decreased. At the family level, TAC treatment caused an increase in the relative abundance of *Rikenellaceae* and *Verrucomicrobiaceae*, but a decrease in the relative abundance of *Clostridiales incertae sedis XIII*, *Lachnospiraceae* and *Ruminococcaceae*.

Overall, our results indicate that TAC administration affects both the α- and β-diversity metrics of the gut bacterial composition. After 5 days of treatment, TAC-treated mice had lower evenness, with a disturbed abundance of specific bacteria.

### ATB-mediated gut microbiota depletion affects TAC PK and reduces TAC blood exposure

Reciprocally, we aimed to study the contribution of the gut microbiota to TAC PK. Current broad-spectrum ATB cocktails are often associated with reduced appetence and must be therefore administered by gavage twice daily to avoid impacting food intake and body weight [37]. As TAC was also administered by gavage daily, for ethical purposes, we therefore evaluated another broad-spectrum ATB cocktail. The association of meropenem, vancomycin, and neomycin administered for 4 days was efficient at reducing bacterial load (~ 100-fold reduction) while having no impact on body weight, food intake, and water consumption (Figure S4).

**Fig. 3:**
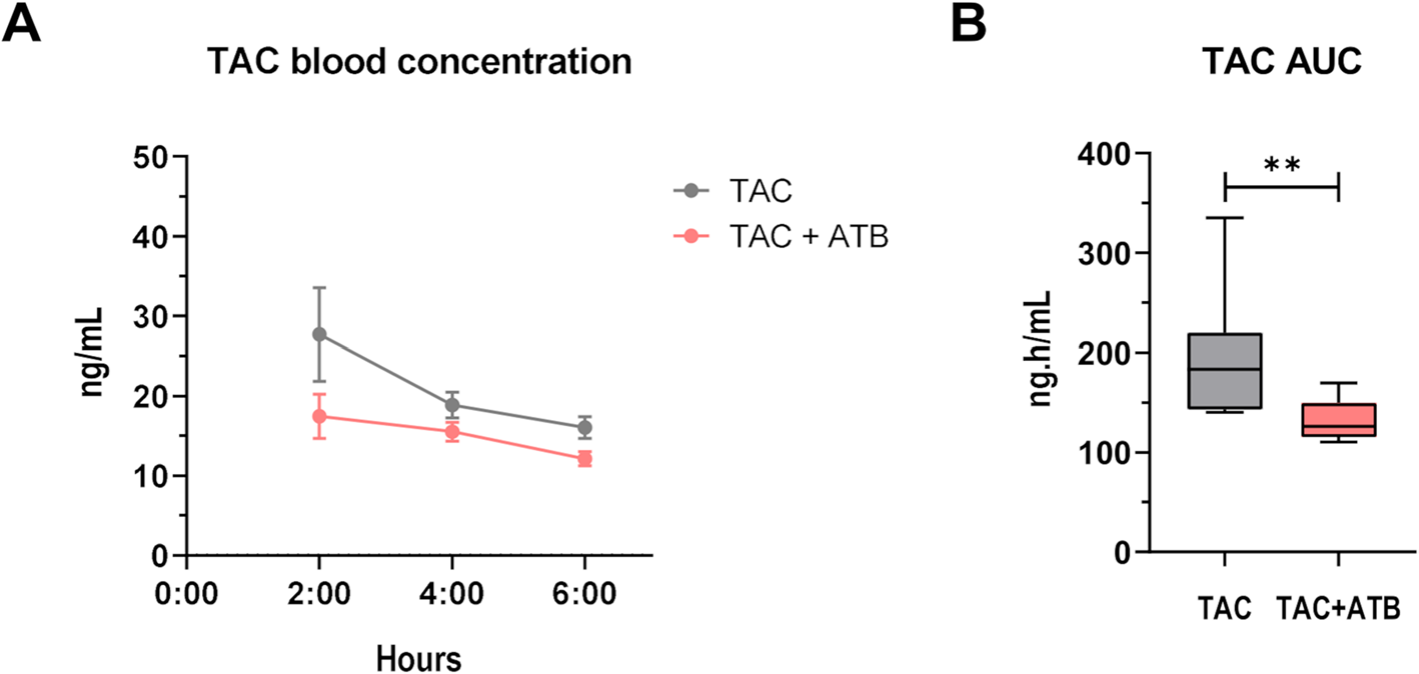
Antibiotic (ATB)-mediated gut microbiota depletion affects tacrolimus (TAC) pharmacokinetics (PK) and reduces TAC blood exposure. **A** TAC blood concentration in control (TAC) or ATB-treated mice (TAC + ATB). **B** TAC area under the curve (AUC) computed with the PK model for both mouse groups (*n* = 10/group). **p < 0.01

Using this ATB cocktail, TAC blood concentrations in control (TAC) and ATB-treated mice (TAC + ATB) were compared at steady-state. ATB-mediated gut microbiota depletion caused a decrease in TAC blood levels at all time points (Fig. 3A). Population PK-derived AUC were then derived to better estimate the whole drug blood exposure. ATB-treated mice showed significantly lower TAC AUC when compared to mice treated with TAC solely (TAC 195.4 vs TAC + ATB 131.1 ng h/mL) (Fig. 3B). Moreover, Fisher’s exact test revealed a significant reduction in the TAC AUC variance of the ATB-treated mice (*p* = 0.003).

These results show that the gut microbiota increases TAC AUC and contributes to at least some extent to the inter-individual variation.

### *Abcb1a* expression in the small intestine is microbiota-dependent and correlates with TAC whole blood exposure

**Fig. 4:**
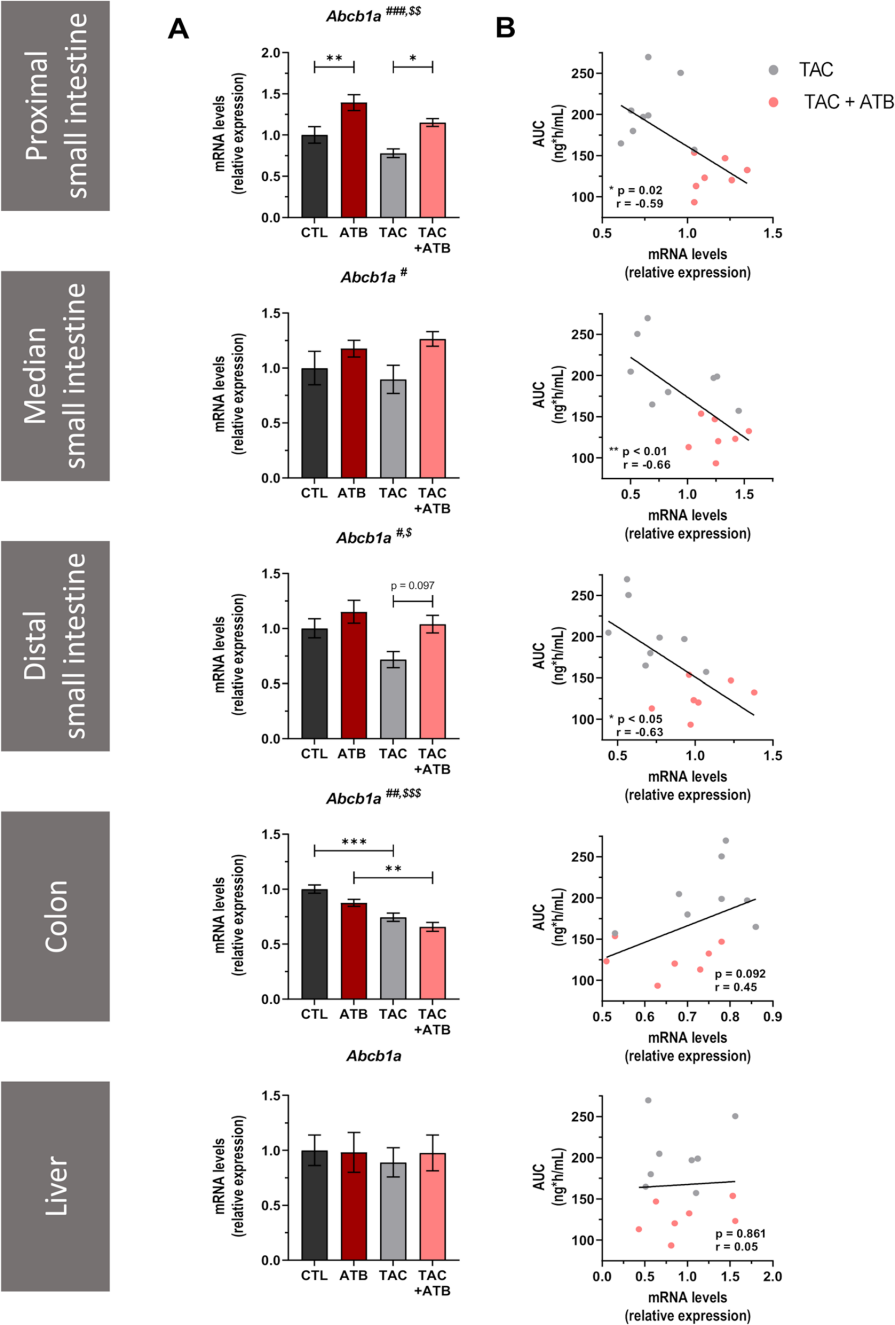
*Abcb1a* expression in the small intestine correlates with tacrolimus (TAC) whole blood exposure. **A** Comparison of the mRNA expression of *Abcb1a* in the proximal, median and distal small intestine; in the colon; and in the liver of control and ATB-treated mice, with or without TAC treatment (*n* = 7–8/group). ^#^significant ATB effect; ^$^significant TAC effect; ***p < 0.001, **p < 0.01, and *p < 0.05. **B** Pearson’s correlation between TAC area under the curve (AUC) and *Abcb1a* mRNA expression level in the different tissues

To decipher the underlying mechanisms by which bacteria affect TAC blood levels, we analysed the mRNA expression levels of the key TAC-processing genes (*Cyp3a11*, *Cyp3a13*, and *Abcb1a*) in the intestinal and hepatic tissues (Fig. 4A, Figure S5). Two-way ANOVA analyses were performed to evaluate significant ATB-mediated gut microbiota reduction effect and/or TAC effect (indicated on the graphs by # and $, respectively). In the distal small intestine and in the liver, *Cyp3a11* was significantly decreased in ATB-treated mice, whereas *Cyp3a13* was unaffected (Figure S5). Such observation argues against the implication of *Cyp3a11* and *Cyp3a13* in the ability of the gut microbiota to increase TAC blood exposure. Interestingly, ATB-mediated gut microbiota reduction in mice was associated to an increased expression of the efflux transporter *Abcb1a* in the small intestine (Fig. 4A). Besides, *Abcb1a* expression level in the proximal, median and distal segments of the small intestine was significantly correlated with TAC AUC values (Fig. 4B). By contrast, *Abcb1a* expression was significantly reduced in the colon but not affected in the liver upon ATB treatment.

**Fig. 5:**
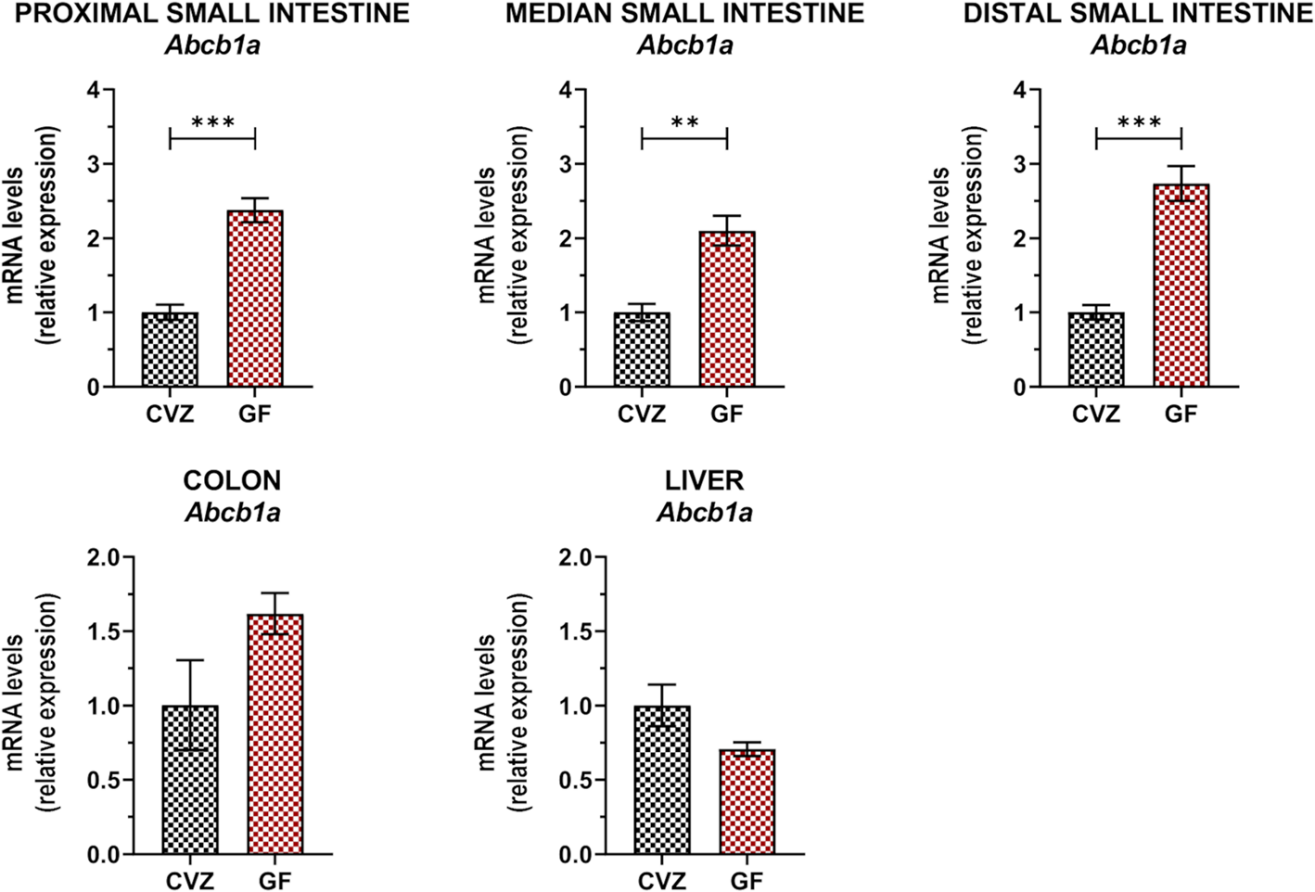
*Abcb1a* expression level is microbiota-dependent in the small intestine. Comparison of the mRNA expression of *Abcb1a* in the proximal, median and distal small intestine; in the colon; and in the liver of conventionalized (CVZ) and germ-free (GF) mice (*n* = 4–5/group). ***p < 0.001 and **p < 0.01

As the ATB used for the microbiota depletion could potentially affect directly *Abcb1a* expression, we used an alternative ATB cocktail (ATB2) on one side and an ATB-independent experimental setting on the other side to confirm the ability of the microbiota to modulate *Abcb1a* expression. The expression of *Abcb1a* in the ileum was increased by 2.1-fold in mice under ATB2 as compared to the controls (Figure S6). In the second approach, we investigated the impact of the conventionalization of germ-free mice with a complex gut microbiota on the expression of *Abcb1a*. In the absence of a gut microbiota, *Abcb1a* expression was increased all along the small intestine as compared to the conventionalized mice (CVZ) (Fig. 5). By contrast, *Abcb1a* expression in the colon and liver was not significantly different between CVZ and GF mice.

Altogether, these observations certify that the expression of *Abcb1a* in the small intestine is microbiota-dependent, and this effect is site-specific. Consequently, we reasoned that the increased *Abcb1* expression constitutes a likely explanation for the reduced TAC blood levels in ATB mice, as a higher intestinal efflux capacity would lead to less intestinal absorption.

### ABCB1A inhibition reverses the gut microbiota effect on TAC PK

**Fig. 6:**
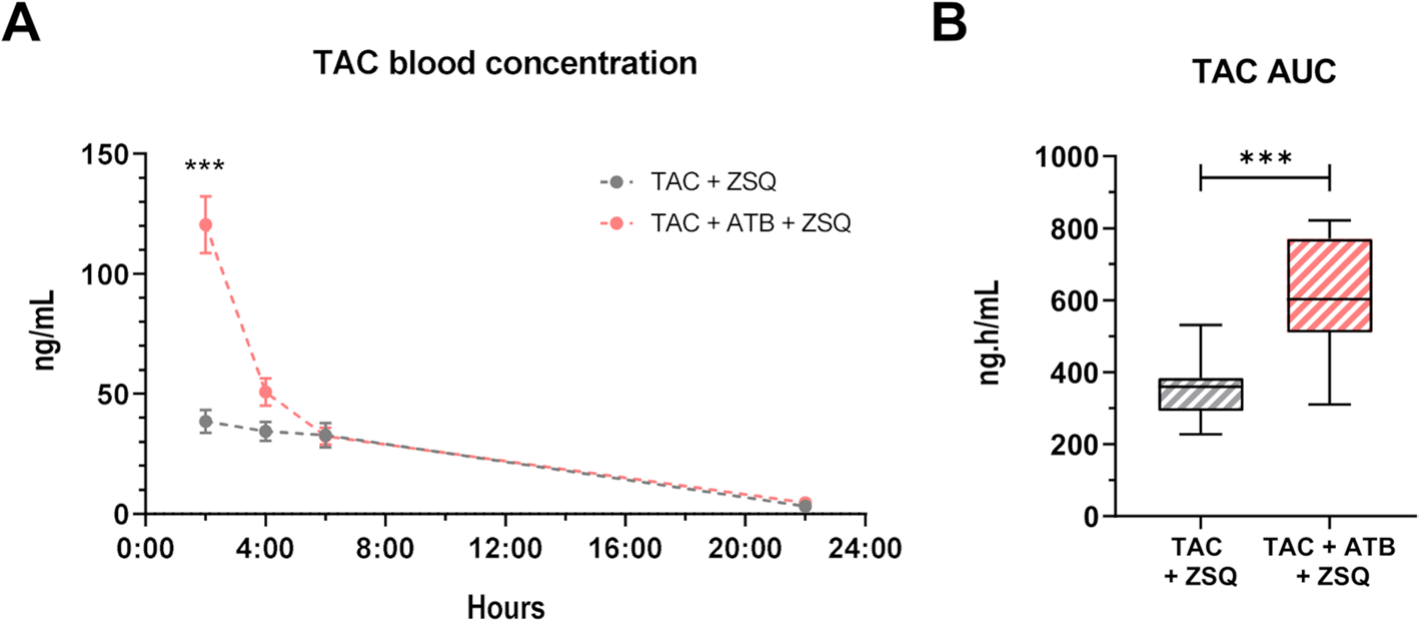
ABCB1A inhibition reverses the gut microbiota effect on tacrolimus (TAC) pharmacokinetics (PK). **A** TAC blood concentration in control (TAC) or antibiotic (ATB)-treated (TAC + ATB) mice under ABCB1 inhibitor, zosuquidar (ZSQ). ***p < 0.001. **B** TAC area under the curve (AUC) derived from the PK model in TAC + ZSQ or TAC + ATB + ZSQ mice (*n* = 10*–*11/group). ***p < 0.001

To mechanistically decipher the contributing role of the gut microbiota to TAC PK through ABCB1, we measured TAC blood levels in mice harbouring a normal or ATB-reduced microbiota, treated for 5 days with TAC, and receiving the last day, 15 min before TAC administration, a potent ABCB1 inhibitor, zosuquidar (ZSQ, also known as LY335979) [38]. Under ZSQ supplementation, ATB-treated mice showed a 75% increased TAC whole blood exposure compared to control mice (TAC + ZSQ 353.8 vs TAC + ZSQ + ATB 617.5 ng.h/mL) (Fig. 6A, B) showing that the functional inhibition of ABCB1A counteracted the effect of the gut microbiota on TAC PK. An increased inter-individual variance in the TAC AUC was observed in TAC + ZSQ + ATB mice versus TAC + ZSQ mice (Fisher’s exact test, *p* = 0.04).

**Fig. 7:**
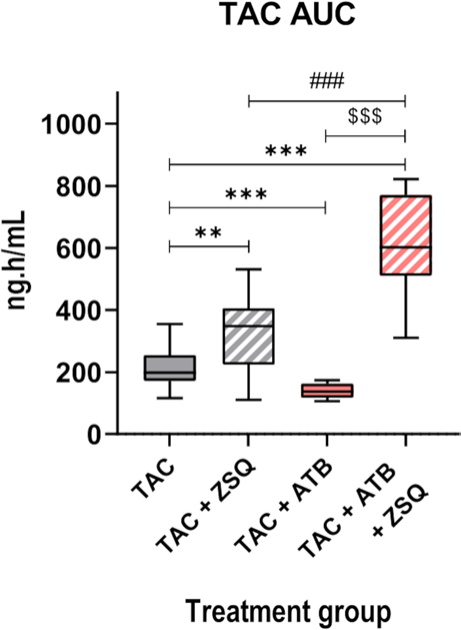
Modulation of ABCB1A efflux activity by the gut microbiota explains differences in tacrolimus (TAC) blood exposure. TAC area under the curve (AUC) derived from the PK model in mice under control (TAC), zosuquidar (TAC + ZSQ), antibiotics (TAC + ATB), or ZSQ with ATB (TAC + ATB + ZSQ) conditions (*n* = 10–36/group). ***p < 0.001, **p < 0.01; ^###^*p* < 0.001 (TAC + ZOSU vs TAC + ATB + ZOSU); ^$$$^*p* < 0.001 (TAC + ATB vs TAC + ATB + ZOSU)

Using our population PK model, all the single effects observed in our previous experiments were recapitulated in a pooled analysis (Fig. 7). In comparison to TAC solely group, ATB treatment significantly decreased TAC AUC, whereas ZSQ supplementation was associated with a significant increase in TAC AUC supporting its ABCB1A inhibition activity. ZSQ effect was even more important in mice under ATB treatment corroborating the implication of ABCB1A in the ATB-mediated effect. In addition, the effect of ABCB1A activity on the intra-group variability was still observed: as the ABCB1A activity decreases (TAC + ATB > TAC > TAC + ZSQ ≈ TAC + ATB + ZSQ), the variability increases as evaluated by the Brown-Forsythe’s test of equality of variance (*p* < 0.001).

Altogether, these results formally demonstrate that the modulation of TAC PK by the gut microbiota is mediated through ABCB1. These data also support a link between ABCB1 expression/activity, TAC exposure and inter-individual variations.

### Polar bacterial metabolites impact the transcriptional regulation of ABCB1, but not directly its functionality

The gut microbiota is an important source of metabolites and compounds. As we postulated that the impact of the gut microbial depletion on ABCB1 expression/functionality might be mediated through bacterial metabolites, we exposed cells to faecal water (FW).

**Fig. 8:**
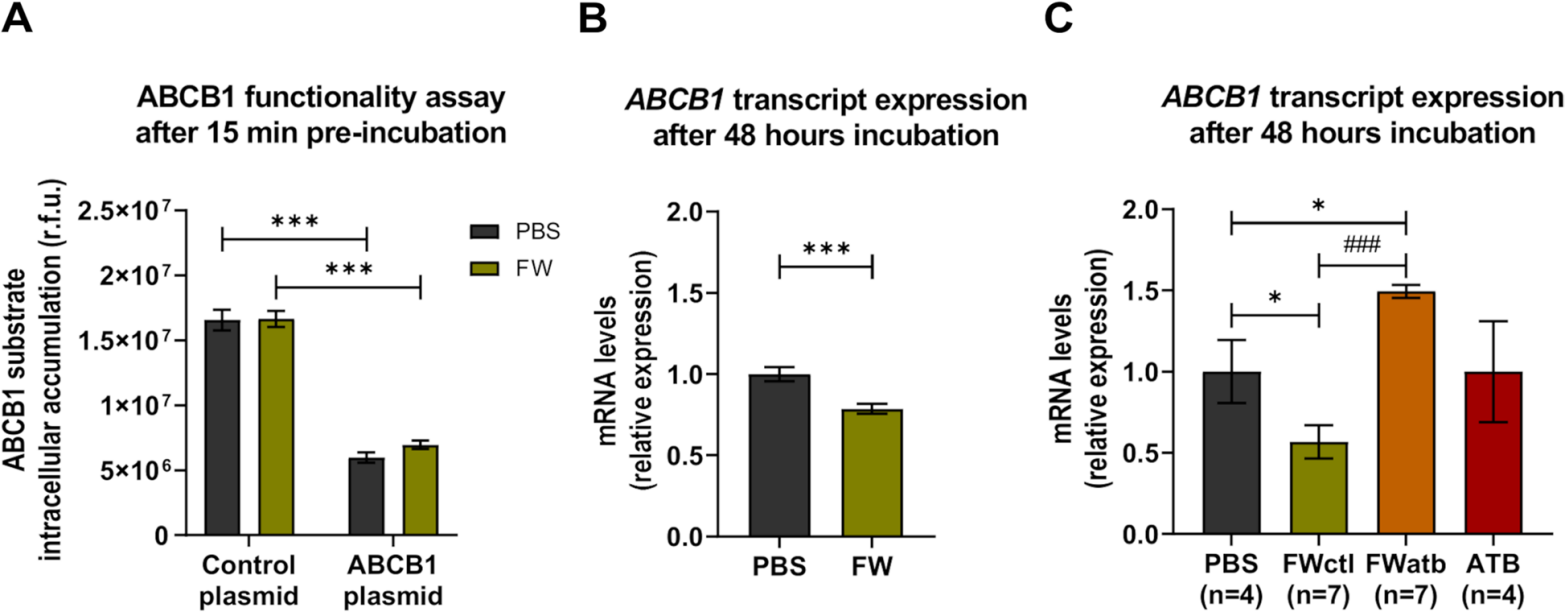
Polar bacterial metabolites impact the transcriptional regulation of *ABCB1*, but not directly its functionality. **A** ABCB1 substrate (rhodamine 123) intracellular accumulation (in relative fluorescent units, r.f.u.) with or without a 15 min pre-exposition to mouse faecal water (FW) in control plasmid or in ABCB1-transfected cells (*n* = 6/group, *N* = 2). ***p < 0.001. **B** Comparison of the mRNA expression of *ABCB1* in control Caco-2 cells (PBS) or cells exposed to mouse FW for 48 h (*n* = 3/group, *N* = 5). ***p < 0.001. **C** Comparison of the mRNA expression of *ABCB1* in control Caco-2 cells (PBS) or cells exposed for 48 h to FW from untreated mice (FWctl), or to FW from ATB-treated mice (FWatb), or to antibiotics (ATB) (*n* indicated on the figure, for FW *n* = 7 biological replicates). *p < 0.05 and.^###^*p* < 0.001 (paired *t*-test between FWctl and Fwatb)

First, we tested the impact of FW on the intrinsic ABCB1 efflux functionality. The intracellular accumulation of rhodamine 123 (Rh123), a fluorescent-specific substrate of ABCB1 [39, 40], was performed in HEK293 recombinant cells. Rh123 accumulation was compared between control cells (Control plasmid) expressing low basal levels of ABCB1 and stably transfected cells overexpressing ABCB1 (ABCB1 plasmid), pre-exposed or not to FW for 15 min (Fig. 8A). As expected and as reflected by the significantly lower intracellular fluorescence in ABCB1 overexpressing cells, ABCB1 overexpressing cells accumulated significantly less Rh123 when compared to control cells under normal condition (PBS). However, FW pre-exposure did not significantly affect the functionality of ABCB1 as similar intracellular fluorescence values were measured in both cell lines.

Then, we investigated whether FW exposure affects *ABCB1* mRNA expression in an in vitro human colon epithelial cancer cell model expressing physiological levels of transporters, i.e. Caco-2 cells [41]. Caco-2 cells are generally acknowledged as a suitable model for the study of drug intestinal absorption/transport [40, 42, 43]. Cells exposed for 48 h to FW from SPF mice had significantly lower mRNA expression level of *ABCB1* as compared to cells treated with PBS (PBS) (Fig. 8B). This result shows that exposing Caco-2 cells to polar metabolites arising from mouse faeces can modulate *ABCB1* expression.

To evaluate if this response was bacteria-dependent, we looked at the effect of FW when mice had an ATB-mediated gut microbiota depletion. We exposed Caco-2 cells for 48 h to FW of the same mice before (FWctl) and under ATB treatment (FWatb) (Fig. 8C). FW in normal condition (FWctl) significantly reduced *ABCB1* mRNA expression when compared to the control condition (PBS). Conversely, FWatb significantly increased *ABCB1* mRNA expression, as compared to the vehicle-treated cells (PBS) and to the FWctl. Additionally, exposing cells directly to the ATB cocktail (ATB) did not affect significantly *ABCB1* expression, proving that the FWatb effect was not directly mediated by the ATB.

Altogether, these data confirm our in vivo findings that intestinal bacteria repress *ABCB1* expression and suggest that this effect is mediated through bacteria-derived polar metabolites and/or compounds. However, these metabolites do not directly affect the intrinsic ABCB1 efflux functionality.

### CAR may mediate the effect of the microbiome on *ABCB1* expression

**Fig. 9:**
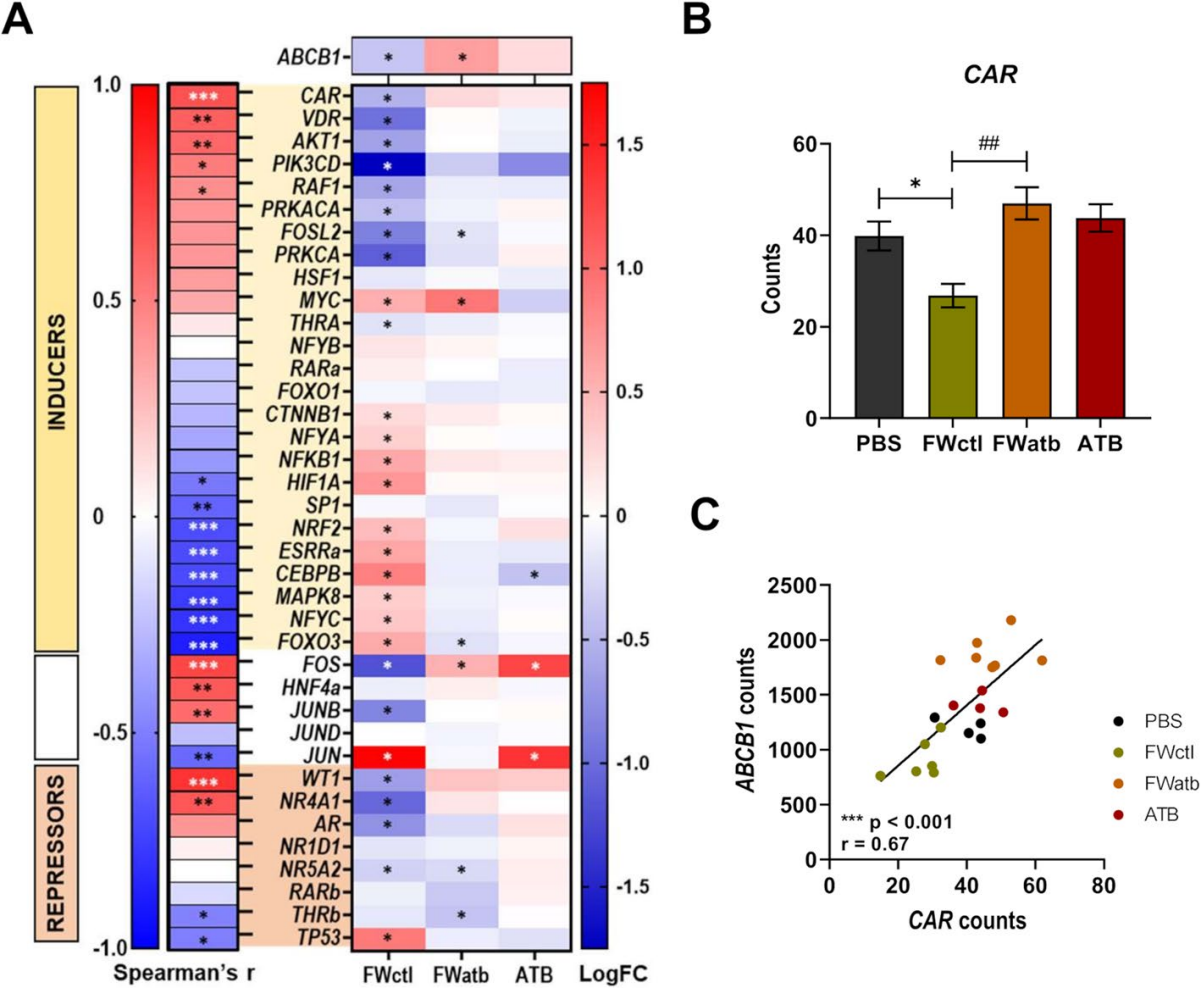
The nuclear receptor CAR may mediate the effect of the microbiome on *ABCB1* expression. Whole transcriptome analysis of control Caco-2 cells (PBS) or cells exposed for 48 h to FW from untreated mice (FWctl), or to FW from ATB-treated mice (FWatb), or to antibiotics (ATB) (*n* = 4–7/group). **A** Analysis of the transcription factors (TF) known to have inductive (INDUCERS, yellow box), variable (white box) or repressive (REPRESSORS, orange box) effects on *ABCB1* expression. On the left, *rho* value is represented for Spearman’s correlation between the expression of *ABCB1* and each of the TF. ***p < 0.001, **p < 0.01, *p < 0.05. On the right, heatmap showing the log twofold change (LogFC) for each condition compared to the control (PBS). *adjusted *p* < 0.05. **B** Comparison of the mRNA expression of *CAR* in these different conditions. *p < 0.05, ^###^*p* < 0.001 (paired *t*-test between FWctl and Fwatb). **C** Spearman’s correlation between *CAR* and *ABCB1* mRNA expression levels in these conditions

The transcriptional regulation of ABCB1 is complex and involves many transcription factors (TF) [44]. To further determine by which intracellular signalling pathway(s) the microbiome affects *ABCB1* expression, we performed a whole transcriptome analysis of the Caco-2 cells exposed to PBS, FWctl, FWatb or ATB. 1736 genes were differentially expressed between FWctl and FWatb conditions, and 4367 genes were found to be significantly correlated with *ABCB1*. We focused our attention on the TF previously identified by Chen et al. [45] and by Foley et al. [46] that modulate the intestinal expression of *ABCB1.* For each of those TF (with either inductive, repressive or controversial effect on *ABCB1* expression), we computed the correlation between their expression and *ABCB1* mRNA levels and, in parallel, we examined their relative expression profile in the different conditions as compared to the controls (Fig. 9A). The Constitutive Androstane Receptor (CAR) had the strongest significant correlation, with a profile that mirrors the most the profile of *ABCB1* in those cells (Fig. 9B, C). The reduction in FWctl condition was also coherently associated with the change in other TF (i.e. VDR, AKT1, RAF1 and TP53). Altogether, the transcriptome analysis pinpoints CAR as a potential mediator of the effect of the microbiome on *ABCB1* expression.

### mRNA expression of CAR correlates with *Abcb1a *levels in mouse intestine

**Fig. 10:**
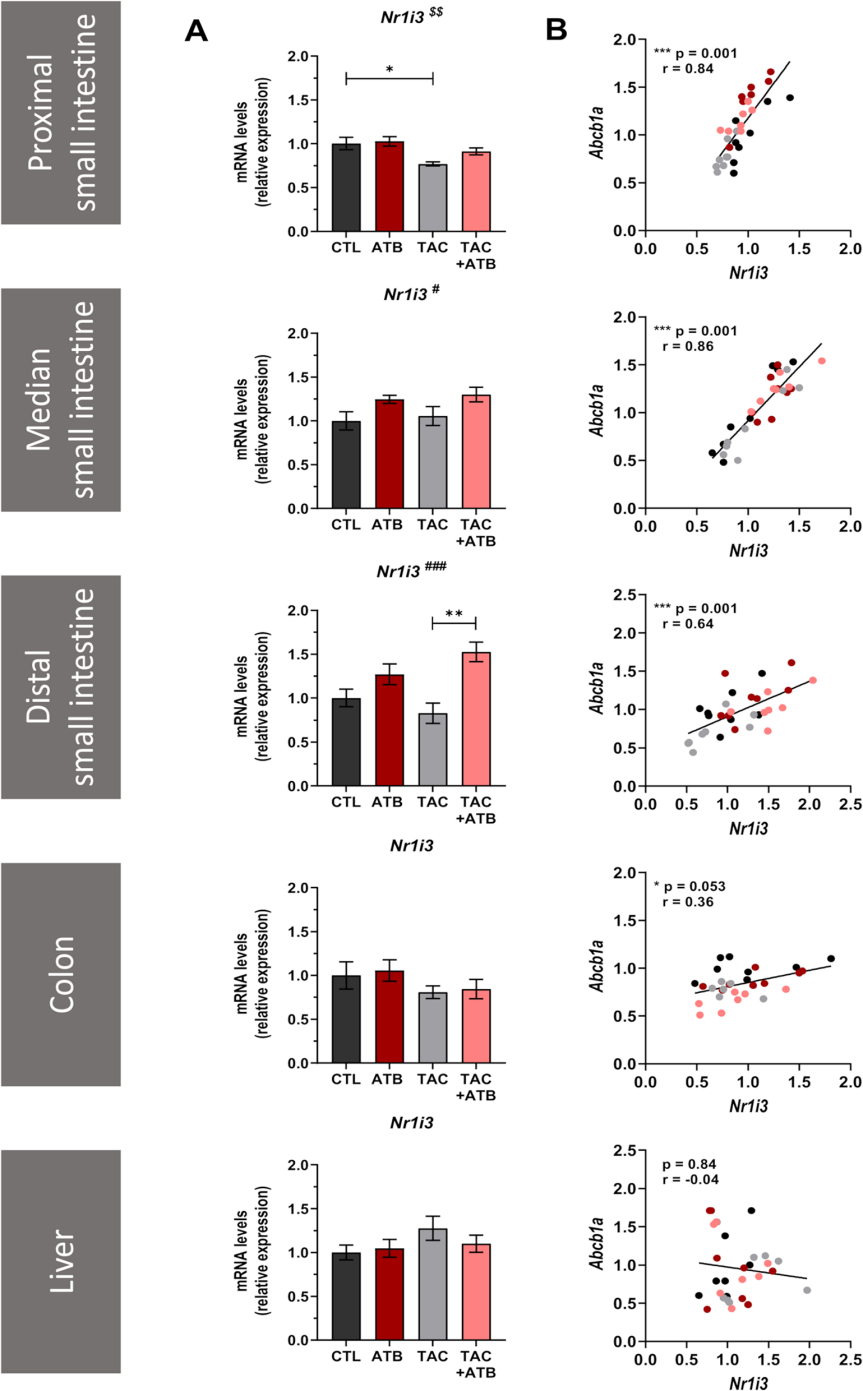
mRNA expression of CAR correlates with *Abcb1a* levels in mouse intestine. In mice, the transcription factor CAR is encoded by the *Nr1i3* gene. **A** Comparison of the mRNA expression of *Nr1i3* in the proximal, median and distal small intestine; in the colon; and in the liver of control and ATB-treated mice, with or without TAC treatment (*n* = 7–8/group). ^#^significant ATB effect; ^$^significant TAC effect; ***p < 0.001, **p < 0.01, and *p < 0.05. **B** Spearman’s correlation between *Nr1i3* and *Abcb1a* mRNA expression levels in the different tissues

Finally, as our in vitro results indicated that CAR (encoded by *Nr1i3* in mice) may be involved in the microbial regulation of *ABCB1* expression, we measured its expression in mice and determined the correlation with *Abcb1a* (Fig. 10A, B). ATB treatment increased *Nr1i3* expression in the median and distal small intestine and strong significant positive correlations between *Abcb1a* and *Nr1i3* expression were observed all along the intestinal tract. This correlation was, by contrast, not observed in the liver. Similarly, *Nr1i3* expression was increased in GF mice as compared to CVZ, and significant correlations to *Abcb1a* expression were observed in the small intestine (Figure S7). Altogether, these in vivo results are coherent with our previous in vitro observations and further support the involvement of CAR in the modulation of intestinal *ABCB1* expression by polar bacterial metabolites.

## Discussion

**Fig. 11:**
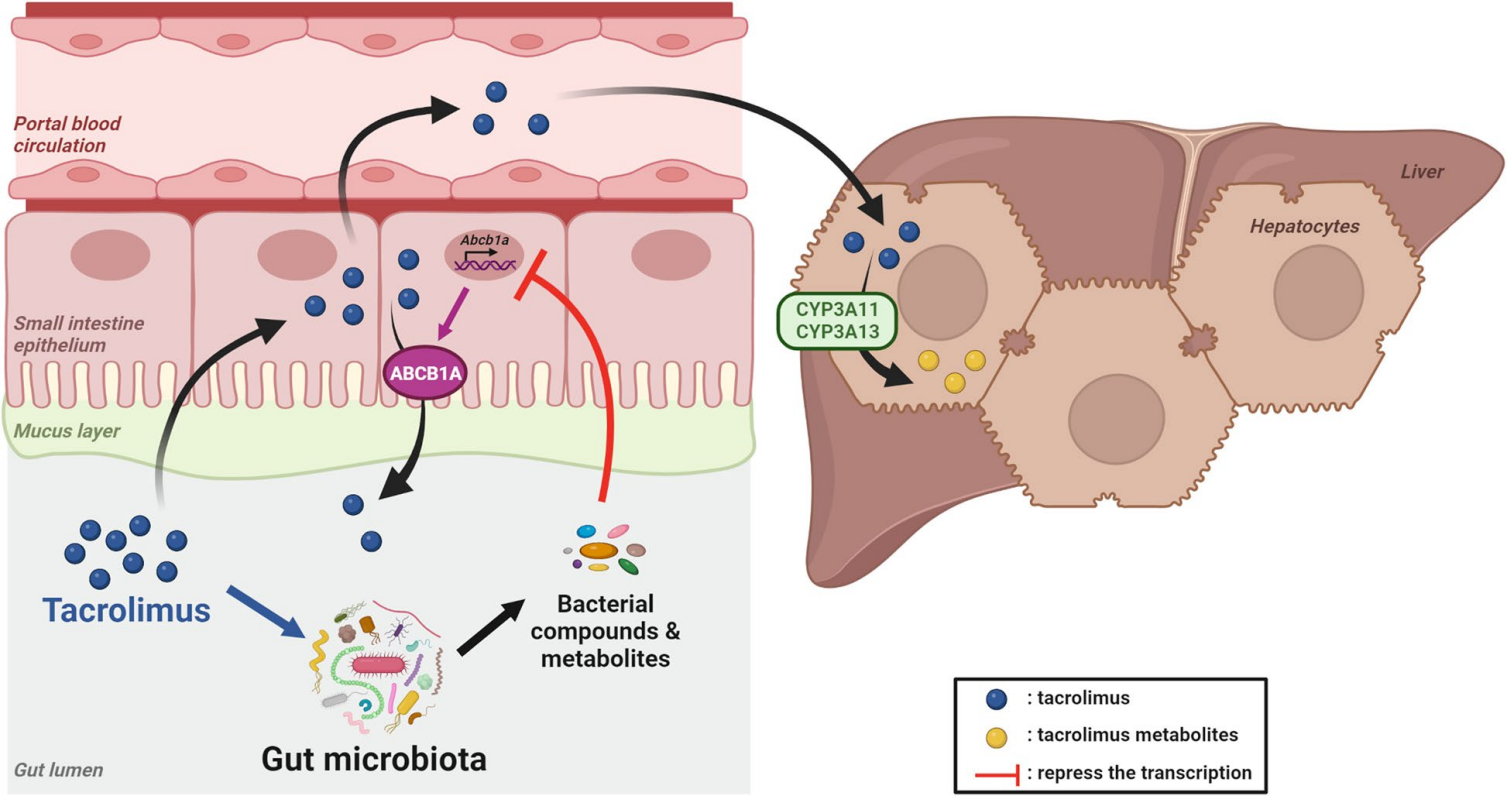
Schematic summary of the bidirectional interactions between tacrolimus (TAC) and the gut microbiota. After oral administration, TAC diffuses passively in the small intestine but the efflux transporter ABCB1A (encoded by *Abcb1a* in mice) limits its absorption. Some of the drug reaches the blood circulation and is then metabolized in the liver by CYP3A enzymes (encoded by *Cyp3a11* and *Cyp3a13* in mice). In the lumen, the gut microbiota produces compounds/metabolites. Some of them have a repressive effect on *Abcb1a* expression (red arrow), resulting in less efflux activity. Moreover, TAC impacts the gut microbiota composition (blue arrow). Created with BioRender.com

The clinical use of TAC is currently a real challenge due to its narrow therapeutic index and its high inter- and intra-individual PK variability. In the present study, we explored the contribution of the gut microbiota to this PK variability, as well as the reciprocal effect of TAC oral administration on the gut microbiota composition. We developed a robust and clinically relevant model of TAC oral administration in mice and we showed that TAC rapidly induces changes in the faecal microbiota composition. We also highlighted that the gut microbiota modulates TAC intestinal absorption. Indeed, polar microbial compounds cause the reduction of *ABCB1* expression in the small intestine, contributing to higher inter-individual variability. Lastly, our analyses emphasize that the underlying intracellular signalling mechanism(s) may involve the modulation of the nuclear receptor CAR by these polar bacterial metabolites (Fig. 11).

As we aimed to study the bidirectional interaction between the gut microbiota and TAC PK, we first evaluated the impact of TAC administration on the gut microbiota composition. We found out that both the α-diversity (mainly the evenness) and the β-diversity of the intestinal microbiota were altered, while the relative abundance of specific taxa was affected. However, the compositional nature of gut microbiota composition measurements makes impossible to distinguish an absolute increase in a specific taxon under TAC treatment from a relative increase due to the relative decrease of other taxa [47]. Like in the present study, previous studies in mice also reported an increased relative abundance of *Alistipes* and decreased relative abundances of *Clostridiales*, *Lachnospiraceae* and *Ruminoccocaceae* under TAC [48, 49]. However, they did not observe change in the relative abundance of *Oscillibacter*, while *Akkermansia* and *Verrucomicrobioaceae* were either unaffected Zhang et al. [48] or decreased [49]. The lack of control for the excipient effect when using trade formulation could explain these discrepancies between our results and these previous studies. Similar to our findings, most human studies show a decrease of the evenness under TAC-based therapy [19, 50]. Contrary to human clinical studies that consistently showed an increase in *Firmicutes* and *Proteobacteria* after transplantation in patients treated with TAC [19, 50], we did not observe any significant changes in those phyla after TAC administration in mice. However, existing human studies looking at the gut microbiota changes in renal transplantation are considering both the transplantation and the immunotherapy effect (reviewed in Winichakoon et al. [51]). Indeed, in patients, it is highly challenging to isolate the specific effect of TAC administration from other inherent patient singularities (transplantation surgery, other co-medications and pathologies, dietary habits, graft function, etc.). Forslund and colleagues were able to disentangle in other contexts the specific effect of medication on the gut microbiota from the effect ensuing from the disease status, with integrated multi-omics analyses and large cohorts [52, 53]. In our context, one way to go to decipher TAC-specific effect would be to compare the faecal microbiota of patients with similar treatments, only differing for their dose of TAC.

TAC meets several criteria for a potential contribution of the gut microbiota to its PK, as described by Spanogianopoulos et al., namely an oral administration, a low absorption and a biliary elimination [54]. Accordingly, a direct biotransformation of TAC by intestinal commensal bacteria has been previously reported in an ex vivo setting with high TAC concentrations and extended incubation times [25]. One could thus expect that a reduced intestinal load generated by ATB administration would lead to a diminished bacterial TAC biotransformation and, consequently, higher TAC blood levels. Surprisingly, we observed a decreased TAC whole blood exposure in mice with an ATB-depleted microbiota. Thenceforth, even if the direct contribution of the gut microbiota to TAC metabolism cannot be discarded, its importance in the PK of the drug seems to be minor in vivo. Coherently, a pilot study conducted in 10 kidney transplant recipients demonstrated that, even if the bacteria-derived TAC metabolite is found in patients, it constitutes, at most, only 5% of the parent drug [55].

Beyond direct drug metabolism, the gut microbiota is also known to affect drugs through indirect mechanisms including the modulation of host gene expression [54, 56]. Originally, the first PK components that were discovered as influenced by the gut microbiota are the CYP factors [57, 58]. Consistently, we found out that *Cyp3a11* hepatic expression is reduced under ATB-mediated microbiota depletion. Upon combined administration of ATB and ZSQ, TAC AUC was even more increased in the ATB-treated mice when compared to the control group. ZSQ has been shown in vitro to also inhibit CYP3A (Ki ~ 3.8 μM); its affinity for CYP3A is more than 60 times lower than for ABCB1 (Ki ~ 59 nM) [38]. Consequently, an unspecific inhibitory effect on CYP3A is very unlikely at the administered dose. One possible explanation for this increase is that, with the inhibition of ABCB1A activity by ZSQ, more TAC is absorbed. Consequently, a higher amount of TAC reaches the liver in both the control and ATB-treated mice but leads to a saturation of hepatic metabolism only in ATB-treated mice. Indeed, the lower *cyp3a11* expression in ATB-treated mice reduces their hepatic biotransformation capacity causing CYP3A metabolism saturation when ABCB1A is inhibited. It may explain why TAC blood levels observed in this group are even higher when compared to the group of mice treated with TAC + ZSQ solely.

Thereafter, our results demonstrate that the efflux transporter ABCB1, rather than the CYP, seems to be responsible for the modulation of TAC PK by the intestinal microbiota. Accordingly, we found out that *Abcb1a* expression in all segments of the small intestine, where the drug is mainly absorbed, correlated with TAC AUC. Previous works have shown that *Abcb1a* intestinal expression is indeed altered in GF or ATB-treated mice [15, 43, 59]. However, those studies were not always consistent in their findings, with increase [59], decrease [43] or no change [15] reported. Also, our data show a decrease of *Abcb1a* expression in the colon of ATB-treated mice. In agreement with our findings, in their study, Foley and colleagues also put forward an inductive effect of the microbiota on *Abcb1a* expression in the colon, through the use of ATB-treated and GF mice [60]. Taken together, our results point out a site-specific modulation of *Abcb1a* expression by the gut microbiota, with opposite effects in the upper and distal part of the intestinal tract.

Importantly, we were able to recapitulate the in vivo effect of the depletion of the microbiota on intestinal expression of *ABCB1* in an in vitro setting on human cells, using polar bacterial metabolites. This strongly suggests that in vivo, bacterial metabolites may also drive and be responsible for such effect and that similar mechanisms are involved both in mice and humans. The regulation of *ABCB1* expression is under the control of numerous TF [44]. Among the many TF able to interact with response elements in the *ABCB1* promotor, the nuclear receptors PXR (Pregnane X Receptor), CAR and VDR (Vitamin D Receptor) bind to the steroid xenobiotic receptor element, after heterodimerization with the retinoic X receptor [44]. Some bacterial metabolites are commonly admitted as being ligands of these nuclear receptors. As examples, lithocholic acid, a secondary bile acid, is ligand of PXR and VDR [61], while indole-3-propionic acid, a bacterial metabolite derived from tryptophan, is also a PXR ligand [62, 63]. Foley and colleagues highlighted that some bacterial metabolites (butyrate and secondary bile acids in combination) modulate the expression of numerous TF involved in the regulation of *ABCB1* in the colon [46]. However, by which mechanism(s) polar bacterial metabolites may downregulate *ABCB1* in the small intestine remains speculative at this stage.

Our experiments provide several pieces of evidence suggesting the implication of CAR in the regulation of *ABCB1* by the gut microbiota. First, using an untargeted approach, we found out that *CAR* displayed the strongest significant correlation with *ABCB1*, with a profile that mirrors the most the one of *ABCB1* in an in vitro setting. Similarly, *CAR* expression closely mirrored the expression of *ABCB1* in vivo. Secondly, the induction of *ABCB1* upon ATB is observed in the small intestine, but not in the colon, while *CAR* expression level is much higher in the small intestine than in the colon (our own data and [64]). The differential microbial regulation of *ABCB1* between the two intestinal regions may result from tissue specificities in the pattern of TF expressed and therefore involved. Furthermore, CAR is known to be expressed in Caco-2 cells unlike PXR [65] and CAR was previously identified by Chen and colleagues as a major regulator of *ABCB1* expression in small intestine T cells, unlike PXR or VDR [45]. All the elements combined led us to identify CAR, rather than PXR, as the most likely TF to be involved.

Undoubtedly, the modulation of *ABCB1* expression by the microbiome cannot be restricted to CAR. Among other important TF, the expression profile of VDR, AKT1, FOS and JUNB exhibited a strong positive correlation with *ABCB1* even if these associations were not as strong as observed for CAR. As mentioned above, VDR has been previously shown to induce the intestinal expression of *ABCB1*, notably in vitro following its activation by the lithocholic acid [63]. The role of AKT1, a downstream element of the phosphoinositide-3-kinase (PI3K) cascade, in the upregulation of *ABCB1* is strongly established in cancer models in link with chemotherapeutic resistance mechanism [44]. Focusing on FOS and JUNB, they are, with JUND and JUN, constituents of the transcription factor complex AP-1. The difficulty with these AP-1 components resides in the fact that their effect on *ABCB1* expression is controversial, with both inducing and repressing effects described [44, 46]. Correlation with *ABCB1* expression was positive, negative or absent for *FOS* and *JUNB*, *JUN*, *JUND*, respectively, making the overall interpretation of the AP-1 complex difficult at this stage. Furthermore, we cannot exclude that, beside the transcription factors presented in Fig. 9, some of the 4367 genes associated to *ABCB1* could be involved in the transcriptional intestinal regulation of *ABCB1* through a pathway not yet characterized. Undeniably, further studies will be necessary to completely elucidate the underlying molecular mechanisms.

Our work clearly establishes the contribution of the gut microbiota to TAC PK. The following arguments support our conclusion that the effect reported on *Abcb1a* (mice)/*ABCB1*(human) expression is mediated by the gut microbiota and not resulting from the ATB themselves: (i) a different cocktail of ATB also induced *Abcb1a* expression, (ii) treatment of two intestinal cell lines with ATB did not change *ABCB1* levels (Caco-2 cells, Fig. 8, and LS174T cells, Figure S8), (iii) polar bacterial metabolites from ATB-free mice were able to recapitulate the impact of the gut microbiota on *ABCB1* levels, (iv) GF mice and ATB-treated mice display similar induction of *Nrli3*, potentially responsible for the induction of *Abcb1a*.

Our work also provides a possible explanation for TAC PK variability. As mentioned earlier, identifying factors explaining intra- and inter-individual variabilities in TAC PK remains a major challenge in TAC therapy management and a way to better control immunosuppression in daily practice.

While clinical reports have described important variations in TAC blood levels after ATB [22, 23], TAC high intra-individual variability has been identified as a risk factor for the graft outcome [4, 11]. Importantly, the above-mentioned TAC PK fluctuations observed under antibiotherapy could not be attributed to direct drug-drug interactions (i.e. CYP and/or ABCB1 induction/inhibition). Alternatively, our study indicates that, by unbalancing the gut microbiota and consequently their derived metabolites, antibiotherapy has the potential to affect *ABCB1* expression, as shown here in the small intestine and by Foley et al. in the colon [60], and to further explain the observed PK changes. Consequently, we believe that therapeutic drug monitoring must be strongly intensified during any antibiotherapy, no matter if a direct drug-drug interaction is expected or not. Moreover, dietary habits are an important parameter influencing the gut microbiota composition and its derived metabolome [66]. In renal transplantation, the post-transplant period is conducive to changes in food habits due to the improved renal function resulting in the alleviation of dietary restrictions [67]. Therefore, drastic nutritional changes under stabilized treatment should be considered cautiously and might also be worth to be accompanied by therapeutic drug monitoring. Whether major dietary changes influences TAC intra-individual PK through the gut microbiota is one of the questions opened by our work.

Apart from the intra-individual variability in TAC PK, high inter-individual variability is also observed in clinics and derives from several factors (i.e. genetics, demographics, drug-drug interactions, etc.) [9]. Our work demonstrates that the gut microbiota is also one of those factors. Here, the reduced inter-individual variability in TAC AUC when the gut microbiota is depleted by ATB is particularly interesting. As the gut microbiota varies from one individual to another, it might explain a part of the variability observed in *ABCB1* expression in the small intestine, the site of drug absorption, thereby affecting drug disposition. Translating these observations in clinics will be particularly challenging and deserves to be explored more deeply. Yet, we believe that incorporating a variable assessing the impact of FW from patients on *ABCB1* expression in the algorithm-informed dosage decision has the potential to improve the prediction of TAC appropriate dosage.

The formulation developed for the purpose of this study allowed us to administrate TAC in mice in a reproducible manner. The composition of the vehicle was relatively simple, with only two components. The administration of the vehicle to CTL mice allowed us to control for its own effect on the gut microbiota composition. However, this formulation is not a perfect reflect of the clinical reality where more complex formulations with extended-release liberation are used. Extended-release formulations are developed to reduce the drug release rate, and with such a formulation, the absorption of TAC spreads all along the small intestine. Henceforth, it seems reasonable to think that our observations are expendable to extended-release formulations since we showed that *ABCB1* expression changes occurred in all segments of the small intestine. The mouse model on its own constitutes another limitation of our study. Although it allowed the identification of an indirect mechanism, undescribed so far, for the contribution of the gut microbiota to TAC PK, the integration of our discovery in the current dose prediction algorithm warrants further clinical studies.

As part of the ABC transporter family, the main function of ABCB1 is to protect the body by limiting the disposition of exogenous compounds, including drugs, through its efflux activity [44]. Because ABCB1 limits the intestinal absorption of hundreds of drugs, our study paves the way for compounds within various pharmacological classes [44]. Indeed, the clinical consequence of the gut microbiota effect on *ABCB1* would not be restricted to TAC. It has the potential to be extended to other substrates of ABCB1 for which PK variability hampers their clinical use and requires therapeutic monitoring. For instance, provided additional experimental and clinical validations, the concept that we are bringing forward might be extended to pharmacological classes such as other immunosuppressants (e.g. cyclosporine, sirolimus), antidepressants (e.g. sertraline, citalopram, venlafaxine), antipsychotics (e.g. amisulpiride, risperidone), and antiretrovirals (e.g. dolutegravir, bictegravir). Of notice, the prediction of the relevance of microbial modulation of ABCB1 for the PK of these drugs will need to consider the absorption site of the drug.

## Conclusions

By combining in vitro and in vivo strategies, our preclinical study allowed us to highlight the bidirectional interactions between the immunosuppressive drug TAC and the gut microbiota. We show that TAC administration can affect the gut microbiota composition within a few days of treatment. Reversely, we pinpoint that the gut microbiota affects TAC absorption through the transcriptional down-regulation of the efflux transporter ABCB1 in the small intestine. Polar bacterial metabolites are responsible for this effect, probably via the modulation of TF including but not restricted to CAR, controlling *ABCB1* transcription. We are bringing forward a new mechanistic path linking the gut microbiota, the expression of *ABCB1* and drug PK, here in the specific case of TAC. The perspectives of this work are broad and of clinical importance, considering the high number of other ABCB1 substrate drugs requiring therapeutic drug monitoring.

## Supplementary Information

**Additional file 1:** Supplementary methods. Supplementary tables.

**Additional file 2:** Supplementary Figures.

## Data Availability

The datasets supporting the conclusions of this article are available in the SRA repository (project ID: PRJNA877868), for the raw sequences from 16S rRNA gene sequencing, and in the Gene Expression Omnibus repository (accession number: GSE224034), for the whole transcriptome data.

## Abbreviations

18S rRNA: 18S ribosomal RNA
ABCB1: ATP Binding Cassette Subfamily B, Member 1
ASV: Amplicon sequence variant
ATB: Antibiotic
ATB2: Alternative antibiotic cocktail
AUC: Area under the curve
Caco-2: Human epithelial colorectal adenocarcinoma cells
CAR: Constitutive androstane receptor
CL/F: Apparent clearance
CTL: Control
CVZ: Conventionalized
CYP: Cytochrome P450
FW: Faecal water
GF: Germ-free
HEK293: Human embryonic kidney cells
HPLC: High-performance liquid chromatography
Ka: Absorption rate constant
LogFC: Log twofold change
LS174T: Human colon carcinoma cells
MS: Mass spectrometry
Nr1i3: Nuclear receptor subfamily 1 group I, member 3
PBS: Phosphate-buffered saline
PCA: Principal component analysis
PI3K: Phosphoinositide-3-kinase
PK: Pharmacokinetics
PXR: Pregnane X receptor
qPCR: Quantitative polymerase chain reaction
Rh123: Rhodamine 123
Rpl4: Ribosomal protein L4
SEM: Standard error of the mean
SPF: Specific pathogen-free
T½: Half-life
TAC: Tacrolimus
TF: Transcription factor
V: Volume of distribution
VDR: Vitamin D receptor
ZSQ: Zosuquidar
