# Supplementary material for: Gut microbiome modulates tacrolimus pharmacokinetics through the transcriptional regulation of ABCB1
Source: Microbiome. 2023 Jul 6;11:138. doi: 10.1186/s40168-023-01578-y (PMC10324113; doi:10.1186/s40168-023-01578-y)
Supplement: Supplementary file 2 — Additional file 1: Supplementary methods. Supplementary tables. [file 40168_2023_1578_MOESM1_ESM.docx]

**Gut microbiome modulates tacrolimus pharmacokinetics through the transcriptional regulation of ABCB1**

**Alexandra L. Degraeve^1,2^, Vincent Haufroid^3,4^, Axelle Loriot^5^, Laurent Gatto^5^, Vanessa Andries^6,7,8^, Lars Vereecke^6,7,8^, Laure Elens^1,3,9^, Laure B. Bindels^2,9 #^**

^1^Department of Integrated PharmacoMetrics, PharmacoGenomics and PharmacoKinetics, Louvain Drug Research Institute, Université catholique de Louvain, Brussels, Belgium.

^2^Metabolism and Nutrition Research Group, Louvain Drug Research Institute, Université catholique de Louvain, Brussels, Belgium.

^3^Louvain centre for Toxicology and Applied Pharmacology, Institut de Recherche Expérimentale et Clinique, Université catholique de Louvain, Brussels, Belgium.

^4^Department of Clinical Chemistry, Cliniques Universitaires Saint-Luc, Brussels, Belgium.

^5^Computational Biology and Bioinformatics Unit (CBIO), de Duve Institute, Université catholique de Louvain, Brussels, Belgium.

^6^Department of Internal Medicine and Pediatrics, Ghent University, Ghent, Belgium.

^7^VIB-UGent Center for Inflammation Research, Ghent, Belgium.

^8^Ghent Gut Inflammation Group (GGIG), Ghent, Belgium.

^9^Contributed equally to this work

^#^**Correspondence:** Laure B. Bindels, laure.bindels@uclouvain.be. Avenue E. Mounier, 73, B1.73.11, Brussels, Belgium. ORCID: LBB 0000-0003-3747-3234.

# SUPPORTING INFORMATION

# Supplementary methods

## In vivo studies

*Concentration-time curve design*

24 mice were divided in 2 batches of 12 mice, sampled and euthanatized on 2 consecutive days. On each day, each mouse was allocated to a unique sample scheme of 3 blood samples (e.g., T0h + T2h + T3h) to avoid any bias. Sample schemes are reported in Table S3 and were identical for day 1 (1^st^ batch) and day 2 (2^nd^ batch) and thus only 2 mice had an identical sampling scheme. 12 blood samples were collected at each time point, as depicted below.

*Conventionalisation of germ-free mice*

Conventionalisation was performed by diluting mouse cecal and colonic contents (~0.3 g) in 3 mL reduced phosphate-buffered saline (PBS) with sterile glass microbeads. Intestinal samples were processed in an anaerobic chamber. Tubes were homogenised for 3 min at 30 Hz and then centrifuged at 800 rpm for 1 min to pellet large insoluble material. 0.2 ml of the supernatant was administered by gavage to each GF mouse of the conventionalized (CVZ) group, with 20G disposable plastic feeding tubes. The mouse intestinal samples were obtained from 3 conventionally raised C57Bl6 SPF mice (7 weeks old, Janvier laboratories). The intestinal samples were obtained shortly before colonization and immediately (within 5 min) diluted, then introduced into the GF mice by gavage within 2 h after dilution, under sterile conditions. The remaining solution was frozen at -80°C. The following day, a second gavage was performed to strengthen the colonization. GF mice were colonized at 8-10 weeks old and maintained for 17 days after conventionalisation. At the end of the conventionalisation period, GF and CVZ mice were euthanized and samples were collected for further analyses.

## In vitro studies

*LS174T cell culture and treatment*

Human colon carcinoma (LS174T) cells were cultured in Dulbecco’s Modified Eagle Medium with high glucose and glutamine (Invitrogen, UK) supplemented with 10% (v/v) of foetal bovine serum, 1% (v/v) of antibiotic–antimycotic and 1% (v/v) non-essential amino acids at 37°C in the presence of 5% of CO_2_. Cells were seeded in 6-well plates at a density of 1*10^6^ cells/well, respectively. After 24 h of growth, the medium was renewed and 10% of medium was replaced by either the ATB cocktail itself (ATB), or the vehicle (PBS) as control. After 48 h of exposure, cells were washed and collected for RNA extraction and subsequent gene expression analysis.

*Characterization of HEK293 transfected cell lines*

The generation and characterization of recombinant cell lines have been described in previous work conducted by our lab [1, 2]. Briefly, HEK293 cells were transfected with a plasmid encoding for *ABCB1* (ABCB1 plasmid) or an empty vector (control plasmid). HEK293 cells feature low endogenous levels of expression of ABCB1, ensuring that all ABCB1 expression originates from the transfection process. This model has previously been extensively characterized using flow cytometry, Western blot, and fluorescence microscopy, and validated using reference substrates and inhibitors of ABCB1 [1, 2]. After thawing and at least 7 days of growth in the presence of the selection ATB geneticin (also known as G418) (1 g/l), ABCB1 expression was checked by flow cytometry: 0.5*10^6^ cells were collected by centrifugation and washed twice with ice-cold buffer (PBS, foetal bovine serum 1%, EDTA 1 mM) then re-suspended in buffer supplemented with 10% anti-ABCB1 antibody, 10% isotype control or in buffer with no antibody, and left to incubate for 45 min on ice and in the dark. Finally, the cells were washed with buffer and resuspended before being analysed using a BD FACSVerse flow cytometer (for characterization) or BD FACSAria III (for cell sorting) and the BD FACSuite software (BD Bioscience, USA). Data analyses were carried out in FlowJo v10.6.1.

*ABCB1 functionality assay*

One day before the experiment, 9*10^4^ cells (HEK293) were plated in poly-L-lysine-coated black 96-well plates in complete medium. ABCB1 functionality assay was investigated by using the intracellular accumulation of rhodamine 123 (Rh123), a fluorescent probe of ABCB1 activity. Cells were loaded for 90 min at 37°C with 5 μM of Rh123 in the dark. Cells were pre-incubated for 15 min with either 10% faecal water (FW) or the vehicle (PBS) as control. After incubation with Rh123, the supernatants were discarded. The cells were washed two times with PBS at 4°C and cell lysis was performed with purified H_2_O and ultrasonication by QSonica Q700 sonicator (QSonica LLC., USA). Finally, the intracellular fluorescence of Rh123 was analysed by a fluorimeter SpectraMax i3x (Molecular Devices, USA); excitation wavelength was set at 485 nm and emission at 530 nm. Absence of cytotoxicity of the FW was confirmed using a mitochondrial activity assay (Cell Proliferation Reagent WST-1, Roche, Switzerland) following the manufacturer’s instructions.

## Pharmacokinetic analysis

The principle of population pharmacokinetics (popPK) resides in the integration of all information gathered on the PK of a drug product, whatever the dosing regimen or sampling scheme. While classical PK analysis has inherent problems such as the influence of sampling scheme and the need of a rich sampling strategy, the management of missing samples or dosing compliance, popPK offers advantages. The PopPK modeling approach allows pooling of sparse data collected in many subjects to estimate mean PK parameters along with between-subjects variabilities and to test the covariate effects on these quantitated variabilities, no matter what the study design/sample scheme/co-treatment/dose/regimen. Such a modeling approach has already proved its relevance in other comparable animal studies [3].

PK measurements per time points used to feed our model consisted of 12 C_0h_, 12 C_1h_, 86 C_2h_, 12 C_3h_, 87 C_4h_, 87 C_6h_, 55 C_22h_ (99 mice in total, covering 7 time points).

## Gut microbiota analysis

*DNA extraction and total bacteria quantification*

To assess the impact of ATB on the bacterial load, genomic DNA was extracted from faeces using the QIAamp DNA Stool Mini Kit (Qiagen, Germany) according to manufacturer’s instructions, with the addition of a bead-beating step. To assess the impact of TAC on gut microbiota composition, genomic DNA was extracted from faeces following the protocol Q described by Costea et al [4]. This protocol uses a QIAamp DNA Stool Mini Kit (Qiagen, Germany), including a bead-beating step. Treatment with RNAse A was performed (10 mg/ml, Thermo Fisher Scientific, USA). DNA concentration was determined using a NanoDrop 2000 (Thermo Fisher Scientific, USA).

Absolute quantification of the total bacteria (carried out for the validation of the ATB cocktail as well as for the gut microbiota analysis) was performed by quantitative PCR using the primers Bacteria Universal P338f (ACTCCTACGGGAGGCAGCAG) and P518r (ATTACCGCGGCTGCTGG) [5]. PCR was performed with a QuantStudio3 (Applied Biosystems, USA) using SYBR Green (GoTaq® qPCR mix, Promega, USA) for detection. All samples (0.1 ng/µl) were run in duplicate in a single 96-well reaction plate. Final concentrations were as follow: cDNA 2 µl/25 µl, primers 300 nM, and SyberGreen mix 1X (MeteorTaq DNA polymerase, dNTP, RT buffer, MgCl_2_ 4 mM, SYBR® Green I, ROX passive reference and stabilizers, as provided by the manufacturer). Thermocycling conditions were as follow: initiation step at 95°C 2 min; cycling stage at 95°C 30 s, 60°C 30 s, 72°C 30 s, 40 cycles; melt curve stage at 65°C 20 s, increment of 0.1°C every 1 s until reaching 95°C. Threshold was manually adjusted to reach the linear range of the log-fluorescent curves and CT values were determined using the QuantStudio software v1.4.3 (Applied Biosystems, USA). Absolute quantification was achieved through the inclusion of a standard curve (performed in duplicate) on each plate generated by diluting DNA from pure culture of *L. acidophilus* NCFM (five-fold serial dilution). Cell counts were determined by plating and expressed as “colony-forming unit” (CFU) before DNA isolation.

*16S rRNA gene sequencing – raw data generation*

Amplicon sequencing of the microbiome was done at the University of Minnesota Genomics Center. Briefly, the V5-V6 region of the 16S rRNA gene was PCR-enriched using the primer pair V5F_Nextera (TCGTCGGCAGCGTCAGATGTGTATAAGAGACAGRGGATTAGATACCC) and V6R_Nextera (GTCTCGTGGGCTCGGAGATGTGTATAAGAGACAGCGACRRCCATGCANCACCT) in a 25 μl PCR reaction containing 5 μl of template DNA, 5 μl of 2X HotStar PCR master mix, 500 nM of final concentration of primers and 0.025 U/μl of HostStar Taq+ polymerase (Qiagen Germany). PCR-enrichment reactions were conducted as follow, an initial denaturation step at 95°C for 5 min followed by 25 cycles of denaturation (20 s at 98°C), annealing (15 s at 55°C), and elongation (1 min at 72°C), and a final elongation step (5 min at 72°C). Next, the PCR-enriched samples were diluted 1:100 in water for input into library tailing PCR. The PCR reaction was analogous to the one conducted for enrichment except with a KAPA HiFi Hot Start Polymerase concentration of 0.25 U/μl, while the cycling conditions used were as follows: initial denaturation at 95°C for 5 min followed by 10 cycles of denaturation (20 s at 98°C), annealing (15 s at 55°C), and elongation (1 min at 72°C), and a final elongation step (5 min at 72°C). The primers used for tailing are the following: F-indexing primer AATGATACGGCGACCACCGAGATCTACAC[i5]TCGTCGGCAGCGTC and R-indexing primer CAAGCAGAAGACGGCATACGAGAT[i7]GTCTCGTGG GCTCGG, where [i5] and [i7] refer to the index sequence codes used by Illumina. The resulting 10 μl indexing PCR reactions were normalized using a SequalPrep normalization plate according to the manufacturer’s instructions (Life Technologies, USA). 20 μl of each normalized sample was pooled into a trough, and a SpeedVac was used to concentrate the sample pool down to 100 μl. The pool was then cleaned using 1X AMPureXP beads and eluted in 25 μl of nuclease-free water. The final pool was quantitated by QUBIT (Life Technologies, USA) and checked on a Bioanalyzer High-Sensitivity DNA Chip (Agilent Technologies, USA) to ensure correct amplicon size. The final pool was then normalized to 2 nM, denatured with NaOH, diluted to 8 pM in Illumina’s HT1 buffer, spiked with 20% PhiX, and heat denatured at 96°C for 2 minutes immediately prior to loading. A MiSeq 600 cycle v3 kit was used to sequence the pool. Raw sequences can be found in the SRA database (project ID: PRJNA877868).

*16S rRNA gene sequencing - bioinformatics*

Subsequent bioinformatics analyses were performed *in-house* as previously described [6]. Initial quality filtering of the reads was performed with the Illumina Software, yielding an average of 51 929 pass-filter reads per sample. Quality scores were visualized with the FastQC software (http://www.bioinformatics.babraham.ac.uk/ publications.html), and reads were trimmed to 220 bp (R1) and 200 bp (R2) with the FASTX-Toolkit (http://hannonlab.cshl.edu/fastx_toolkit/). Next, reads were merged with the merge-illumina-pairs application v1.4.2 (with P = 0.03, enforced Q30 check, perfect matching to primers which are removed by the software, and otherwise default settings including no ambiguous nucleotides allowed) [5]. The UPARSE pipeline implemented in USEARCH v11 [7] was used to further process the sequences. Amplicon sequencing variants (ASV) were identified using UNOISE3 [8]. Such method infers the biological sequences in the sample prior to the introduction of amplification and sequencing errors, and distinguishes sequence variants differing by as little as one nucleotide [9]. The analysis allowed the identification of 1 087 ASV. ASV were identified using the RDP database v16. Taxonomic prediction was performed using the *nbc_tax* function [10], an implementation of the RDP Naive Bayesian Classifier algorithm [11]. α-diversity indexes were calculated using QIIME [12] on the rarefied ASV table (rarefaction was performed using Mothur v1.32.1 [13] to avoid large disparities in the number of sequences and a subset of 23 259 reads was randomly selected for all samples).

*16S rRNA gene sequencing – biostatistics*

Unrarefied data were filtered to select for a minimum abundance of 0.01% and a minimal prevalence of 35% in all samples. Principal component analysis (PCA) was performed on CLR-transformed data [14] using the *pca* function in the *mixOmics* R package [15], followed by Permutational Multivariate Analysis of Variance (PERMANOVA), performed per sampling day, using the *adonis* function in the *vegan* R package [16]. The CLR transformation consists in a centered log ratio transformation and allows transforming compositional data into a Euclidian space. A pseudo-count equal to half the minimal value found in the filtered dataset was applied prior the CLR transformation [17]. The PERMANOVA allows to evaluate the explanatory power of TAC treatment. For microbiota data, normality was not inspected for every taxa. Therefore, significantly impacted families and genera over time within each group were identified using Friedman tests with Dunn’s *post hoc* tests, whereas Mann-Whitney U-tests were used to compare CTL and TAC groups after 5 days of treatment. Stacked barplots for phylum, family and genus at day 5 of treatment were generated using the R packages *ggplot2* [18] and *reshape2* [19]. Unrarefied data were filtered to present only taxa with a minimum abundance of 0.01% and a minimal prevalence of 35%. Low abundance taxa and unassigned taxa were aggregated in the “Others” category.

## Gene expression analysis

*Cellular whole transcriptome analysis*

For cellular whole transcriptome analysis, Caco-2 RNA samples were treated with the DNA-*free*^TM^ DNA removal kit (Thermo Fisher Scientific, USA) and with the RNasin Ribonuclease Inhibitor (Promega, USA) according to the manufacturers’ instructions. The quality and quantity of the RNA samples were evaluated using a 2100 Bioanalyzer System (Agilent Technologies, USA). All RIN values were greater than 9, supporting RNA integrity. RNA samples were sequenced after ribosomal RNA depletion using a 2 × 150 paired end configuration on a NovaSeq 6000 instrument (Macrogen, Netherlands). Raw sequence data were generated from Illumina TruSeq Stranded Total RNA library. Fastq files were processed using a standard RNAseq pipeline. Read quality control was performed using FastQC software v0.11.8. Then Trimmomatic software v0.38 [20] was used to remove low quality reads and HISAT2 software v2.1.0 [21] to align reads to the human genome (GRCh38). Gene expression levels were evaluated using the featureCounts software from subread v2.0.0 [22] and Ensembl Homo_sapiens.GRCh38.105.chr.gtf annotation file. Differential expression analyses were performed with DESeq2 Bioconductor package v1.36.0 [23] on R, after filtering to remove genes with a read count > 5 in less than 3 samples. Visualisation of all samples in a PCA plot revealed the presence of one outlier in the FWctl group and this sample was excluded from further analyses. Log 2 fold change (LogFC) were generated for each condition as compared to the control (PBS) and for FWatb as compared to FWctl. P-values were adjusted with the Benjamini and Hochberg procedure and were considered as significant when lower than 0.05. Differentially expressed genes between FWctl and FWatb conditions were defined as having a significant adjusted p-value and an absolute LogFC superior to 1. Spearman's correlation was used to assess correlations between the expression of all genes and ABCB1. Adjusted p-values were computed with Benjamini and Hochberg correction for multiple testing and were considered as significant when lower than 0.05.

We then focused for downstream analyses on transcription factors of interest identified based on a literature search and after selection of the transcription factors with a mean count of at least 15 reads across all samples. Spearman's correlation was used to assess correlations between the expression of the transcription factors and ABCB1. The RNA sequencing dataset generated and analysed for this study can be found in Gene Expression Omnibus (accession number: GSE224034).

## References

1. Dessilly G, Elens L, Panin N, Capron A*, et al.* ABCB1 1199G>A genetic polymorphism (Rs2229109) influences the intracellular accumulation of tacrolimus in HEK293 and K562 recombinant cell lines. PLoS One. 2014;9(3):e91555.

2. Dessilly G, Panin N, Elens L, Haufroid V*, et al.* Impact of ABCB1 1236C > T-2677G > T-3435C > T polymorphisms on the anti-proliferative activity of imatinib, nilotinib, dasatinib and ponatinib. Scientific Reports. 2016;6(1):29559.

3. Zimmermann M, Zimmermann-Kogadeeva M, Wegmann R, Goodman AL. Separating host and microbiome contributions to drug pharmacokinetics and toxicity. Science. 2019;363(6427):eaat9931.

4. Costea PI, Zeller G, Sunagawa S, Pelletier E*, et al.* Towards standards for human fecal sample processing in metagenomic studies. Nat Biotechnol. 2017;35(11):1069-76.

5. Eren AM, Vineis JH, Morrison HG, Sogin ML. A filtering method to generate high quality short reads using illumina paired-end technology. PLoS One. 2013;8(6):e66643.

6. Pötgens SA, Thibaut MM, Joudiou N, Sboarina M*, et al.* Multi-compartment metabolomics and metagenomics reveal major hepatic and intestinal disturbances in cancer cachectic mice. J Cachexia Sarcopenia Muscle. 2021;12(2):456-75.

7. Edgar RC. UPARSE: highly accurate OTU sequences from microbial amplicon reads. 2013;10(10):996-98.

8. Edgar RC. UNOISE2: improved error-correction for Illumina 16S and ITS amplicon sequencing. BioRxiv. 2016:081257.

9. Callahan BJ, McMurdie PJ, Holmes SP. Exact sequence variants should replace operational taxonomic units in marker-gene data analysis. ISME Journal. 2017;11(12):2639-43.

10. Edgar RC. Accuracy of taxonomy prediction for 16S rRNA and fungal ITS sequences. PeerJ. 2018;6:e4652.

11. Wang Q, Garrity GM, Tiedje JM, Cole JR. Naive Bayesian classifier for rapid assignment of rRNA sequences into the new bacterial taxonomy. Appl Environ Microbiol. 2007;73(16):5261-67.

12. Caporaso JG, Kuczynski J, Stombaugh J, Bittinger K*, et al.* QIIME allows analysis of high-throughput community sequencing data. Nat Methods. 2010;7(5):335-6.

13. Schloss PD, Westcott SL, Ryabin T, Hall JR*, et al.* Introducing mothur: open-source, platform-independent, community-supported software for describing and comparing microbial communities. Appl Environ Microbiol. 2009;75(23):7537-41.

14. Gloor GB, Macklaim JM, Pawlowsky-Glahn V, Egozcue JJ. Microbiome datasets are compositional: And this is not optional. Front Microbiol. 2017;8:2224.

15. Lê Cao K, Rohart F, Gonzalez I, Dejean S*, et al.* mixOmics: Omics Data Integration Project. 2017. Available from: <https://CRAN.R-project.org/package=mixOmics>

16. Oksanen J, Simpson G, Blanchet F, Kindt R*, et al.* Vegan: community ecology package. 2023. Available from: <https://github.com/vegandevs/vegan>

17. Mallick H, Rahnavard A, McIver LJ, Ma S*, et al.* Multivariable association discovery in population-scale meta-omics studies. PLoS Comput Biol. 2021;17(11):e1009442.

18. Wickham H. ggplot2: Elegant graphics for data analysis. 2016. Available from: <https://ggplot2.tidyverse.org>

19. Wickham H. Reshaping data with the reshape package. Journal of statistical software. 2007;21:1-20.

20. Bolger AM, Lohse M, Usadel B. Trimmomatic: a flexible trimmer for Illumina sequence data. Bioinformatics. 2014;30(15):2114-20.

21. Kim D, Langmead B, Salzberg SL. HISAT: a fast spliced aligner with low memory requirements. Nat Methods. 2015;12(4):357-60.

22. Liao Y, Smyth GK, Shi W. featureCounts: an efficient general purpose program for assigning sequence reads to genomic features. Bioinformatics. 2014;30(7):923-30.

23. Love MI, Huber W, Anders S. Moderated estimation of fold change and dispersion for RNA-seq data with DESeq2. Genome Biol. 2014;15(12):550-70.

# Supplementary tables

**Table S1. Primer sequences used for mouse tissues**

| **Primers for mouse tissues** | | |
| --- | --- | --- |
| ***Gene*** | **Forward** | **Reverse** |
| *Rpl4* | CAAGAAGACCAAGGAGGCTGT | GGTTTCTCATTTTGCCCTTG |
| *Abcb1a* | ATGCTGCTTGTTTCCGGTTC | CAGGAGCGAATGAACTGACC |
| *Cyp3a11* | CAAACGCCTCTCCTTGCTGT | TATCCCCACTGGGCCAAAATC |
| *Cyp3a13* | TCTGCCTTTCTTGGGGACGA | CCGCCGGTTTGTGAAGGTAGA |
| *Nr1i3* | TTTGCTGGAAGGTGTGAGGT | ATCTGGACCAGTTCTTTCTGCT |

**Table S2. Primer sequences used for human cell lines**

| **Primers for human cells** | | |
| --- | --- | --- |
| ***GENE*** | **Forward** | **Reverse** |
| *18S rRNA* | CGGCTACCACATCCAAGGAA | ATACGCTATTGGAGCTGGAATTACC |
| *ABCB1* | GGTTTGGAGCCTACTTGGTG | GGCTTTGGCATAGTCAGGAG |

**Table S3. Blood sample schemes for the TAC PK profile presented in Figure 1B.**

| Batch | Mice | C_0h_ | C_1h_ | C_2h_ | C_3h_ | C_4h_ | C_6h_ |
| --- | --- | --- | --- | --- | --- | --- | --- |
| 1^st^ | 1 |  |  |  | X | X | X |
|  | 2 |  | X |  | X |  | X |
|  | 3 |  |  | X |  | X | X |
|  | 4 | X | X |  |  |  | X |
|  | 5 | X |  | X |  |  | X |
|  | 6 |  | X | X |  |  | X |
|  | 7 | X |  | X | X |  |  |
|  | 8 |  | X | X | X |  |  |
|  | 9 | X |  |  | X | X |  |
|  | 10 |  | X |  | X | X |  |
|  | 11 | X |  | X |  | X |  |
|  | 12 | X | X |  |  | X |  |
| 2^nd^ | 13 |  |  |  | X | X | X |
|  | 14 |  | X |  | X |  | X |
|  | 15 |  |  | X |  | X | X |
|  | 16 | X | X |  |  |  | X |
|  | 17 | X |  | X |  |  | X |
|  | 18 |  | X | X |  |  | X |
|  | 19 | X |  | X | X |  |  |
|  | 20 |  | X | X | X |  |  |
|  | 21 | X |  |  | X | X |  |
|  | 22 |  | X |  | X | X |  |
|  | 23 | X |  | X |  | X |  |
|  | 24 | X | X |  |  | X |  |
