# Supplementary material for: Gut microbiome modulates tacrolimus pharmacokinetics through the transcriptional regulation of ABCB1
Source: Microbiome. 2023 Jul 6;11:138. doi: 10.1186/s40168-023-01578-y (PMC10324113; doi:10.1186/s40168-023-01578-y)
Supplement: Supplementary file 3 — Additional file 2: Supplementary Figures. [file 40168_2023_1578_MOESM2_ESM.pdf]

Gut microbiome modulates tacrolimus pharmacokinetics through the transcriptional regulation of ABCB1

Alexandra L. Degraeve[1,2], Vincent Haufroid[3,4], Axelle Loriot[5], Laurent Gatto[5], Vanessa Andries[6,7,8], Lars Vereecke[6,7,8], Laure Elens[1,3,9], Laure B. Bindels[2,9 #]

[1]Department of Integrated PharmacoMetrics, PharmacoGenomics and PharmacoKinetics, Louvain Drug Research Institute, Université catholique de Louvain, Brussels, Belgium.
[2]Metabolism and Nutrition Research Group, Louvain Drug Research Institute, Université catholique de Louvain, Brussels, Belgium.
[3]Louvain centre for Toxicology and Applied Pharmacology, Institut de Recherche Expérimentale et Clinique, Université catholique de Louvain, Brussels, Belgium.
[4]Department of Clinical Chemistry, Cliniques Universitaires Saint-Luc, Brussels, Belgium.
[5]Computational Biology and Bioinformatics Unit (CBIO), de Duve Institute, Université catholique de Louvain, Brussels, Belgium.
[6]Department of Internal Medicine and Pediatrics, Ghent University, Ghent, Belgium.
[7]VIB-UGent Center for Inflammation Research, Ghent, Belgium.
[8]Ghent Gut Inflammation Group (GGIG), Ghent, Belgium.
[9]Contributed equally to this work

[#]**Correspondence:** Laure B. Bindels, laure.bindels@uclouvain.be. Avenue E. Mounier, 73, B1.73.11, Brussels, Belgium. ORCID: LBB 0000-0003-3747-3234.

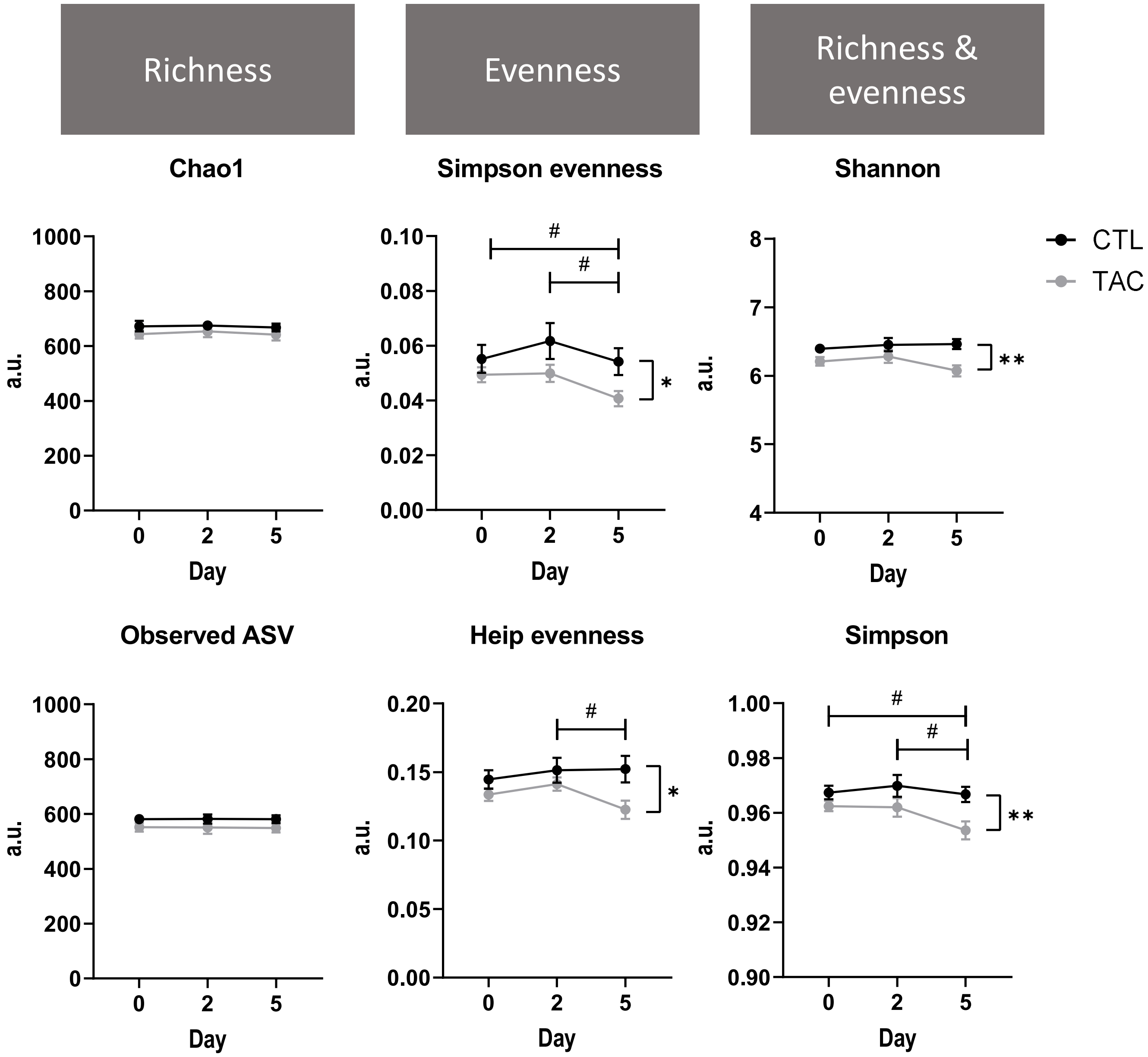

Figure S1. Tacrolimus (TAC) treatment affects the evenness but not the richness of the faecal microbiota. Evolution of α-diversity metrics (in arbitrary units, a.u.) of control (CTL) and TAC-treated mice along the treatment (n = 7-8/group). $^{**}p < 0.01$, $^{*}p < 0.05$ (Mann-Whitney test, CTL vs TAC). $^{\#}p < 0.05$ (Friedman test with Dunn *post hoc* test, TAC repeated-measures).

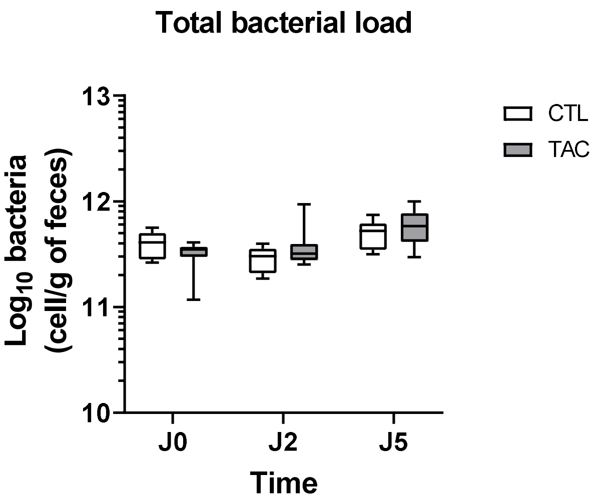

Figure S2. Total bacterial load is unaffected by tacrolimus (TAC) treatment. Evolution of the total bacterial load in faecal samples of control (CTL) and TAC-treated mice along the treatment (n = 7-8/group).

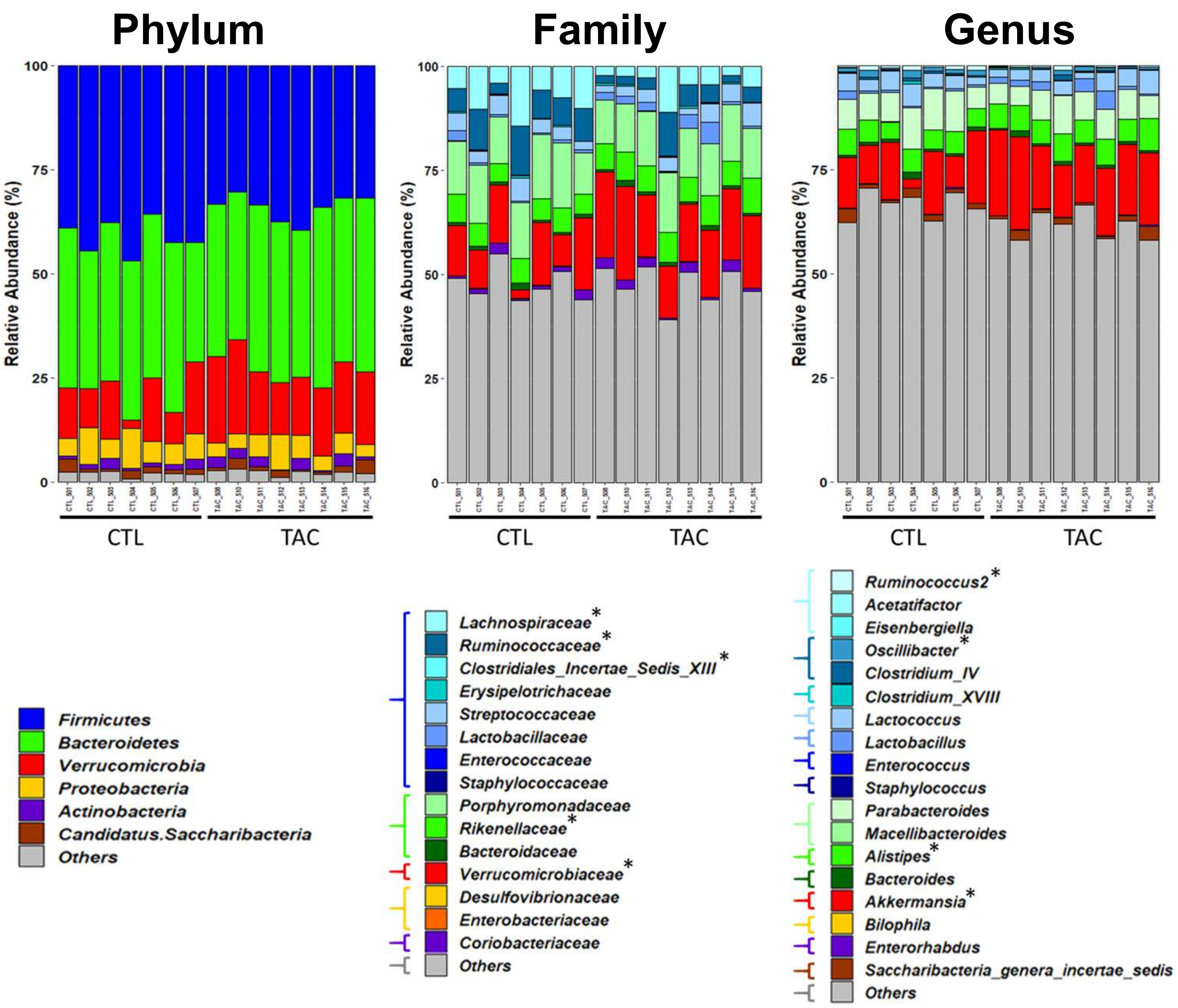

Figure S3. Faecal microbiota composition after tacrolimus (TAC) treatment. Stacked barplots showing the relative abundance of phyla, families, and genera of mice after 5 days of oral gavage with TAC or vehicle (CTL) (n = 7-8/group). * indicated taxa significantly impacted by TAC.

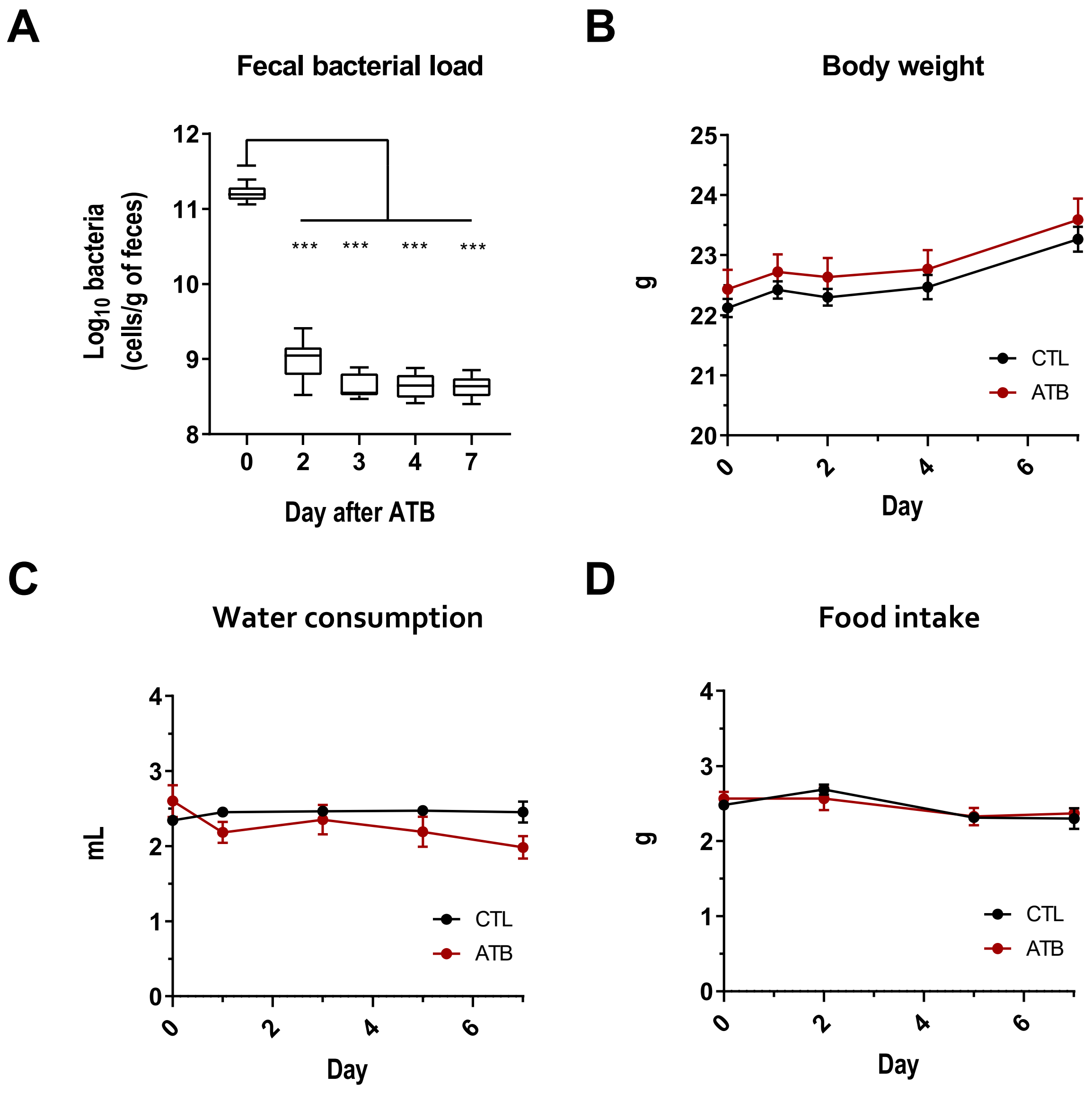

Figure S4. Impact of antibiotic (ATB) treatment on mice monitoring parameters. (A) Faecal bacterial load over ATB treatment. ***p < 0.001. (B-D) The ATB cocktail does not affect the body weight (B), the water consumption (C), or the food intake (D) of the mice (n = 6/group).

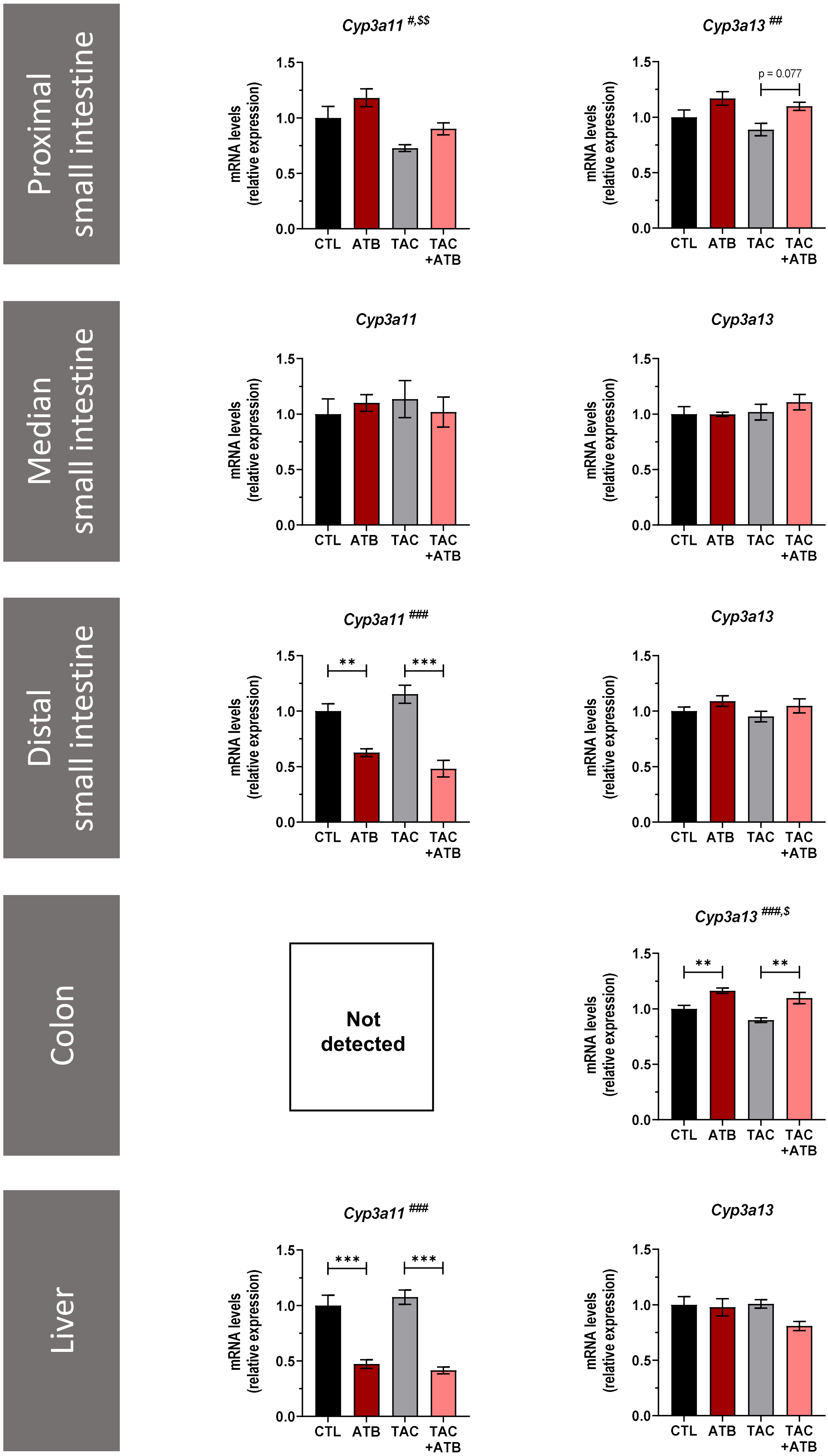

Proximal small intestine
Median small intestine
Distal small intestine
Colon
Liver
Cyp3a11 #,$$
Cyp3a13 ##
p = 0.077
Cyp3a11
Cyp3a13
Cyp3a11 ###
**
***
Cyp3a13
Not detected
Cyp3a13 ###,$
**
**
Cyp3a11 ###
***
***
Cyp3a13
mRNA levels (relative expression)
0.0
0.5
1.0
1.5
CTL
ATB
TAC
TAC +ATB

Figure S5. Antibiotic (ATB) -mediated gut microbiota depletion impacts the mRNA expression of key tacrolimus (TAC) -processing genes. Comparison of the mRNA expression of *Cyp3a11* and *Cyp3a13* in the proximal, median and distal small intestine, in the colon, and in the liver of control and ATB-treated mice, with or without TAC treatment (n = 7-8/group). #significant ATB effect; $significant TAC effect; ***p < 0.001, **p < 0.01 and *p < 0.05.

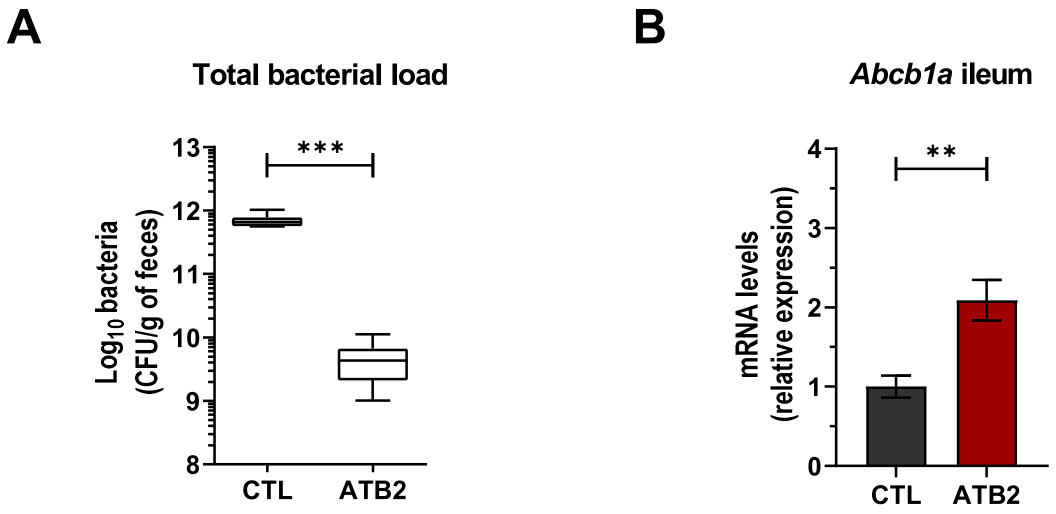

Figure S6. Intestinal *Abcb1a* expression is induced by an alternative antibiotic cocktail (ATB2) used to deplete the gut microbiota. (A) Efficient reduction of the total bacterial load by the ATB2 (neomycin 0.5g/L, ampicillin 1g/L) in BALB/c mice. (B) Comparison of the mRNA expression of *Abcb1a* in the ileum of control and ATB-treated mice (n = 7-8/group). ***p < 0.001 and **p < 0.01.

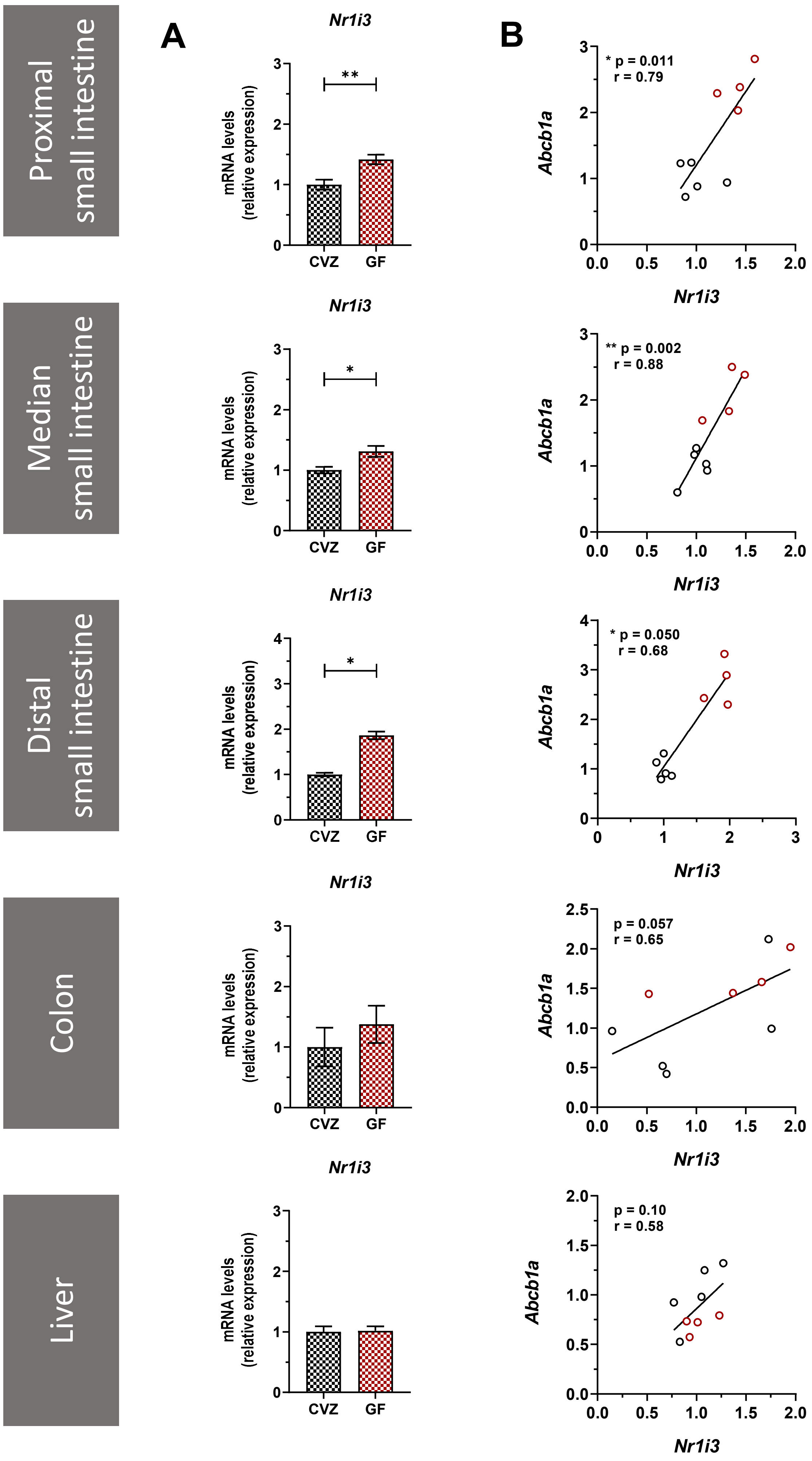

Proximal small intestine
Median small intestine
Distal small intestine
Colon
Liver
A
B
Nr1i3
mRNA levels (relative expression)
CVZ
GF
**
*
*
Abcb1a
* p = 0.011
r = 0.79
** p = 0.002
r = 0.88
* p = 0.050
r = 0.68
p = 0.057
r = 0.65
p = 0.10
r = 0.58

Figure S7. mRNA expression of CAR correlates with *Abcb1a* levels in mouse intestine. In mice, the transcription factor CAR is encoded by the *Nr1i3* gene. (A) Comparison of the mRNA expression of *Nr1i3* in the proximal, median and distal small intestine, in the colon, and in the liver of conventionalized (CVZ) and germ-free (GF) mice (n = 4-5/group). **p < 0.01 and *p < 0.05. (B) Spearman's correlation between *Nr1i3* and *Abcb1a* mRNA expression levels in the different tissues.

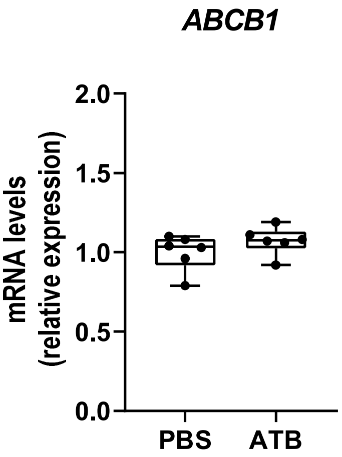

Figure S8. Antibiotics (ATB) do not affect *ABCB1* expression *in vitro*. Comparison of the mRNA expression of *ABCB1* in LS174T cells treated with ATB or vehicle (PBS) for 48 hours (n = 6/group, representative of two independent experiments).
